# Gut dysbiosis in a murine model of cutaneous lupus erythematosus correlates with antigen-specific T cells and antigen-presenting cells in skin

**DOI:** 10.1038/s41598-025-34741-6

**Published:** 2026-01-12

**Authors:** Haley A. Neff, Ümmügülsüm Yıldız-Altay, Nuha Salam, Doyle V. Ward, Dominique Shepard, Zaida G. Ramirez-Ortiz, Jillian M. Richmond

**Affiliations:** 1 Department of Dermatology, UMass Chan Medical School, Worcester, MA USA; 2 Department of Microbiology, UMass Chan Medical School, Worcester, MA USA; 3 Department of Medicine, Division of Infectious Diseases & Immunology, UMass Chan Medical School, Worcester, MA USA; 4 Department of Comparative Pathobiology, Tufts Cummings School of Veterinary Medicine, North Grafton, MA USA; 5 Present Address: Department of Dermatology, Yale University School of Medicine, New Haven, CT USA

**Keywords:** Lupus, Microbiome, Mycobiome, Autoimmune, Shotgun sequencing, Immunology, Microbiology

## Abstract

The commensal organisms constituting the human microbiome are increasingly appreciated to fortify epithelial barriers and modulate host immunity. Dysbiosis of both single strains and communities can contribute to inflammatory conditions. Here, we sought to characterize potential dysbiosis in our inducible mouse model of cutaneous lupus erythematosus (CLE). We hypothesized that gut dysbiosis would occur based on several studies that found lower Firmicutes/Bacteroidetes (F/B) ratios and decreased diversity in systemic lupus erythematosus (SLE) cohorts compared to healthy counterparts, a mouse study that identified Ro60 commensal orthologs that can trigger onset of lupus-like disease, and a study of CLE that identified outgrowth of *Staphylococcus aureus* in the skin. Using whole genome shotgun sequencing, we identified differences in pre- and post-irradiation cohorts, particularly an increase in *Duncaniella*, a decrease in *Prevotella*, and a reduction in alpha diversity following irradiation. Baseline alterations in CLE mice gut bacteria compared to littermate controls were also extant, including trends toward increased *Parabacterides distasonis* and *Bacteroides acidifaciens* in CLE mice. Importantly, we noted an increase in *Phocaeicola sartorii* in CLE mice compared to littermate controls post-disease induction. We examined the mycobiome in our mice and noted a reduction of *Colletotrichum tofieldiae* specifically in CLE mice post-disease induction, and a trend towards increased *Periglandula ipomoeae*. Last, we correlated abundance of genera and species with flow cytometry data obtained from the skin, lymph node and spleen, and identified specific strains that correlated with presence of antigen-specific T cells and different antigen presenting cell populations. Thus, our model exhibits similar changes to other models of lupus-like disease, and our data identify potential novel strains/species that could be modified for CLE and/or SLE treatment such as through generation of probiotics or specific antimicrobial agents.

**Supplementary Information:**

The online version contains supplementary material available at 10.1038/s41598-025-34741-6.

## Introduction

Lupus is a complex and multifactorial disease process attributed to aberrant immune activation against the self, resulting in a heteromorphic symptomatology and several subtypes with varying organ involvement. Most lupus patients present with autoantibodies produced by B cells, autoreactive T cell subsets, and abnormally elevated levels of inflammatory cytokines, hallmarks of both innate and adaptive immune dysregulation^1^. Lupus impacting mucosal tissue or cutaneous tissue is termed cutaneous lupus erythematosus (CLE)^2^, which has an incidence of 4.2–4.3 per 100,000 in the US^3^. CLE and SLE are interrelated diseases: CLE occurs in as many as 80% of SLE patients, and patients that present with isolated CLE can progress to SLE. Adequate control of CLE mitigates the risk of systemic progression^4^, whereas skin damage induced by UV light can exacerbate lupus nephritis^5^.

The microbiome is a relatively new area of investigation for targeted therapy in lupus and other autoimmune diseases^1^. Gut microbiota have been heavily implicated in modulating activities of the immune system, including both innate and adaptive immune system activation and regulation^6^. Microbiota also play a role in the regulation of T cell maturation, and especially the activity of T regulatory cells, which are notably awry in autoimmune diseases^7^.

Dysbiosis, which is defined by a loss of beneficial microbial populations, an increase in deleterious bacterial species, and/or an inappropriately low microbial community diversity, can impair gut and skin barrier function, increasing permeability and thus exposure to the external environment. Dysbiosis has been reported in both lupus-prone mice and human systemic lupus patients^8–10^. Dysbiosis can also trigger autoimmunity directly through molecular mimicry. In the case of lupus, Ro60 orthologues trigger onset of lupus in mice^11^. Few studies have been performed specifically on CLE, though *S. aureus* outgrowth has been characterized in lesional skin^12^. A subsequent study demonstrated that skin *S. aureus* colonization could contribute to SLE, increasing glomerulonephritis and autoantibody deposition, in genetically-prone animals^13^.

Here, we sought to characterize the gut microbiome in our novel B6 mouse model of CLE^14^. This model employs a TLR9KO lupus-prone background, with a keratin 5-driven ovalbumin under the control of a tetracycline response element. This genetic system is inducible and allows for flares. When these recipient mice are injected with Th2-skewed antigen-specific OT2 T cells, mice develop skin lesions that are clinically and histologically similar to human lupus, as well as splenomegaly and positive ANA^15^. We used whole genome shotgun sequencing to analyze fecal pellets from these animals, identifying several bacterial and fungal species that could be targeted for future lupus therapeutic development.

## Results

### The gut microbiome in CLE mice has a lower alpha diversity, and disease induction induces a loss of *Prevotella* and an outgrowth of *Duncaniella*

**Fig. 1:**
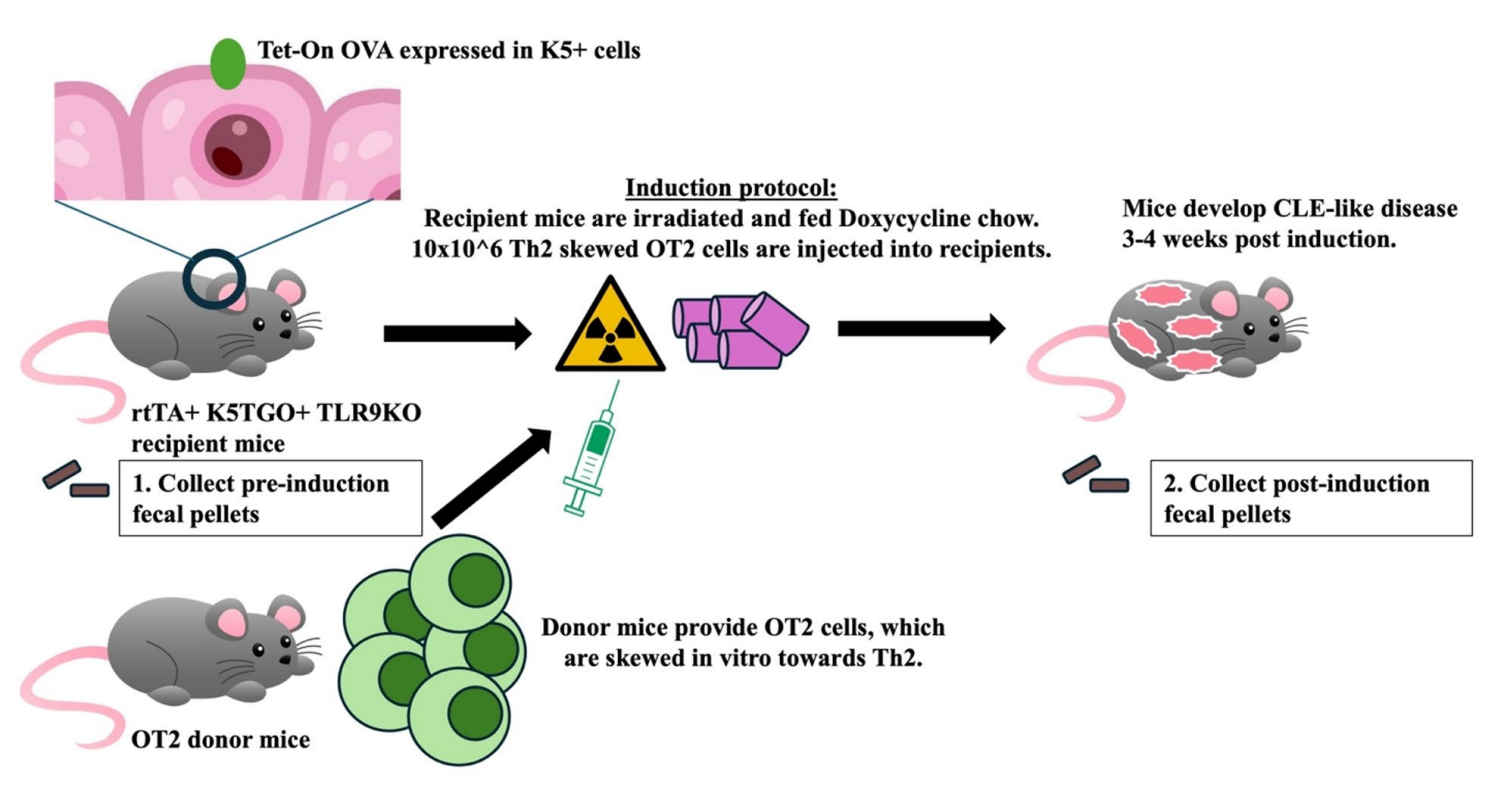
Model induction protocol. Both littermates, who lack the Tet-on OVA promoter, and CLE mice (rtTA + K5TGO + TLR9KO) underwent irradiation and received doxycycline chow. A cohort of littermates (rtTA- and/or K5TGO- genetic controls) also received injections of WT Th2 cells, while others were not injected as negative controls. Pellets were collected from *n* = 4 pre-induction littermates and *n* = 6 pre-induction CLE mice, and *n* = 9 post induction littermates (*n* = 3 injected, *n* = 6 non-injected littermates) and *n* = 9 post-induction CLE mice.

**Fig. 2:**
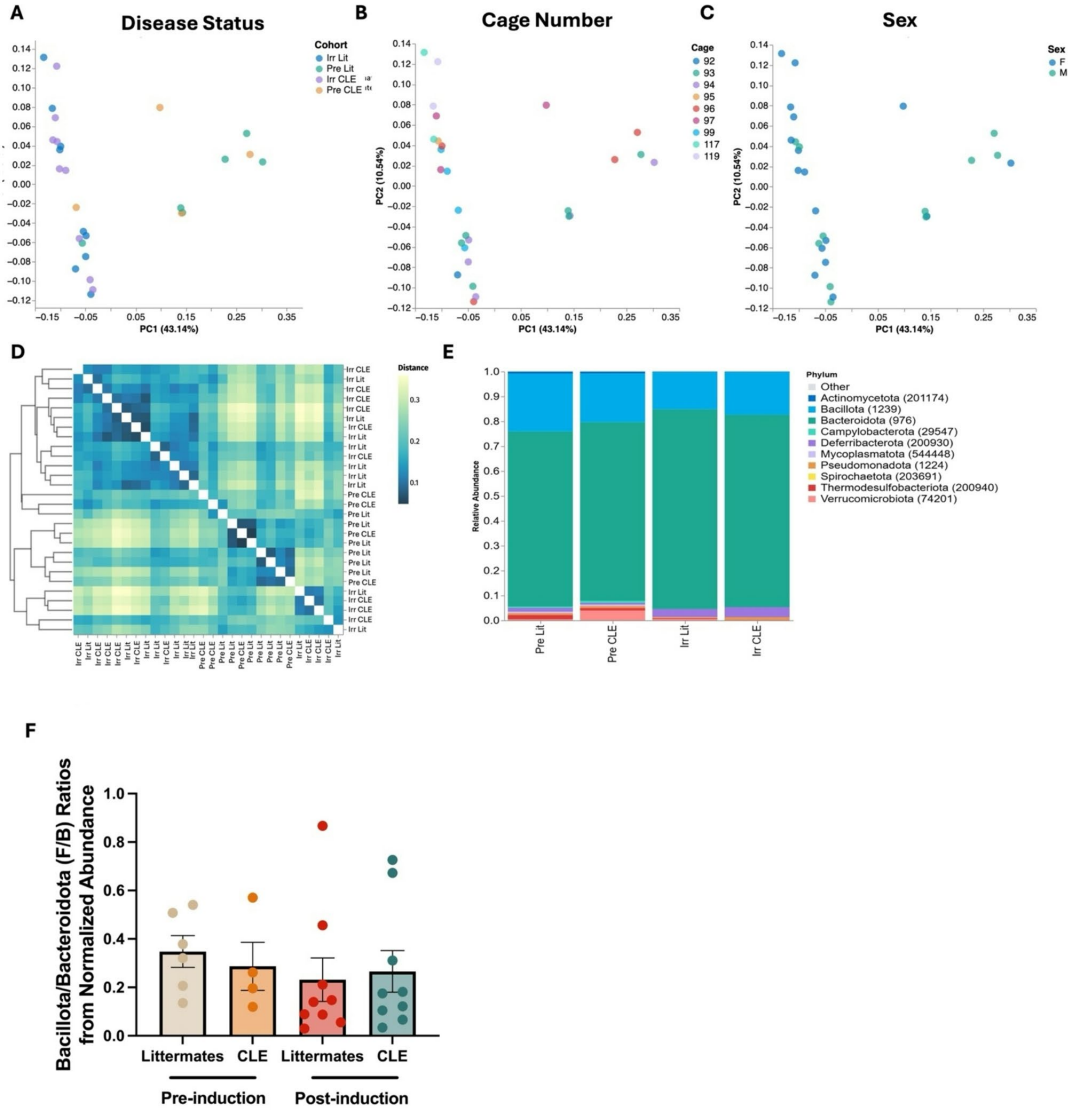
Metrics of relatedness between samples show clustering of irradiated CLE and littermate mice, and no significant difference in F/B ratios. **A**. PCA plot showing distribution by disease status. **B**. PCA plot showing sex differences and **C**. cage differences in the model. **D**. Weighted unique fraction matrix displaying relatedness between samples. **E**. Relative abundance bar graph of the top 10 phyla by disease status demonstrating increased Bacillota (formerly Firmicutes) and reduced Bacteroidota (formerly Bacteroidetes) in CLE mice. Legend displays phyla with NCBI Taxonomic ID number. **F**. F/B ratios calculated from normalized read counts (one-way ANOVA across all groups and t tests for each group pre- vs. post-induction were all ns). Pre-induction mice pooled from *n* = 2 experiments, *n* = 4 pre-induction littermates and *n* = 6 pre-induction CLE mice. Post-induction littermates pooled from *n* = 3 experiments, *n* = 9 post induction littermates and *n* = 9 post-induction CLE mice.

First, we compared fecal microbiome samples from CLE and littermate control mice, both pre- and post-disease induction (Figs. 1 and 2A). Sex and cage number did not significantly cluster on PCA (Fig. 2B, C), therefore samples were pooled for analysis by time (pre- versus post-induction) and genotype status (littermates versus CLE). Based on the weighted unique fraction (unifrac) distance matrix, which takes into account phylogenetic relatedness as well as relative abundance, many pre-induction littermates and pre-induction CLE-prone mice were most similar to each other, though not all post-induction CLE mice were most similar to other post-induction CLE mice, occasionally seeming more similar to their post-induction littermates (Fig. 2D). Bacillota (formerly Firmicutes*) /* Bacteroidota (formerly Bacteroidetes) (F/B) ratios did not significantly differ amongst littermates and their CLE counterparts, though there was a trend towards increased Bacillota and reduced Bacteroidota in CLE mice (Fig. 2E, F). We also observed proportional changes in abundance of specific taxa in irradiated CLE compared to irradiated littermate mice (Fig. S1).

**Fig. 3:**
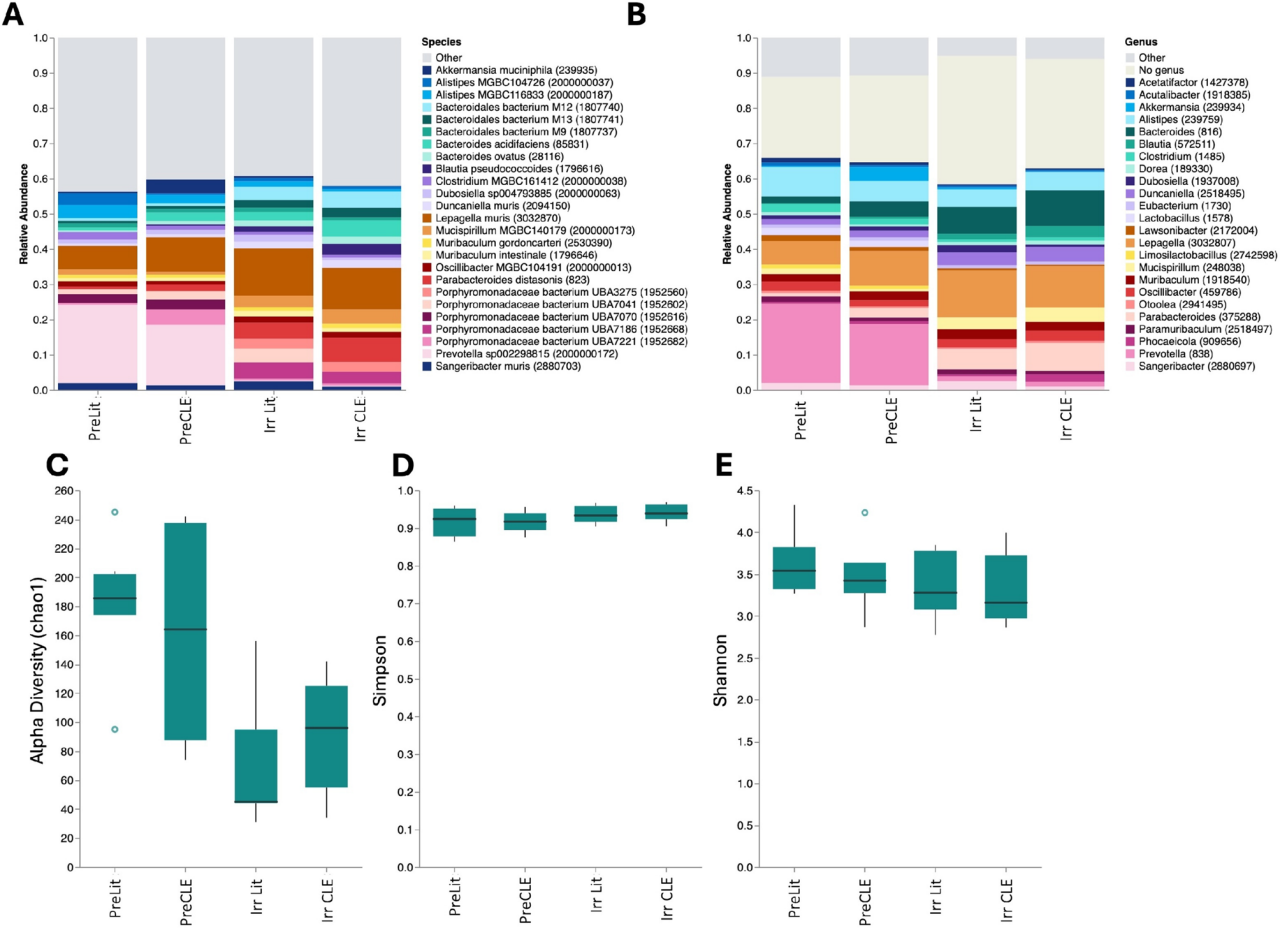
Reduced alpha diversity in CLE mice. **A**. Relative abundance plots of the top 30 most common species and **B**. genera across all samples grouped by disease cohort. Legend displays species or genus name with NCBI Taxonomic ID number. **C**. alpha diversity, **D**. Simpson beta diversity, and **E.** Shannon beta diversity by disease status. Pre-induction mice pooled from *n* = 2 experiments, *n* = 4 pre-induction littermates and *n* = 6 pre-induction CLE mice. Post-induction littermates pooled from *n* = 3 experiments, *n* = 9 post induction littermates and *n* = 9 post-induction CLE mice.

Next, we assessed grouped analyses for pre- and post-induction CLE mice and littermates. Mice with more severe skin lesions tended to have fewer *Prevotella* at the species and genus level (Fig. S2). Examination of differences in pre- versus post-induction revealed a significant increase in *Duncaniella*, *Bacteroidales* and *Lepagella* species, (Fig. 3A, B, S3) and a decrease in *Prevotella* (Fig. 3A, B). CLE mice also had the lowest alpha diversity (Fig. 3C) and trended towards lower beta diversity using Shannon’s Index but not Simpson’s (Fig. 3D, E). Taken together, these data demonstrate that mouse genotype and disease induction process (irradiation, doxycycline administration, T cell injection) were associated with changes in the gut microbiome in the CLE mouse model, and that diseased animals have a lower alpha diversity.

### *P. sartorii* is significantly increased in CLE mice compared to littermates, and *Bacteroides, Parabacteroides, Duncaniella* and *Blautia* increase with disease whereas *Alistipes* decreases

**Fig. 4:**
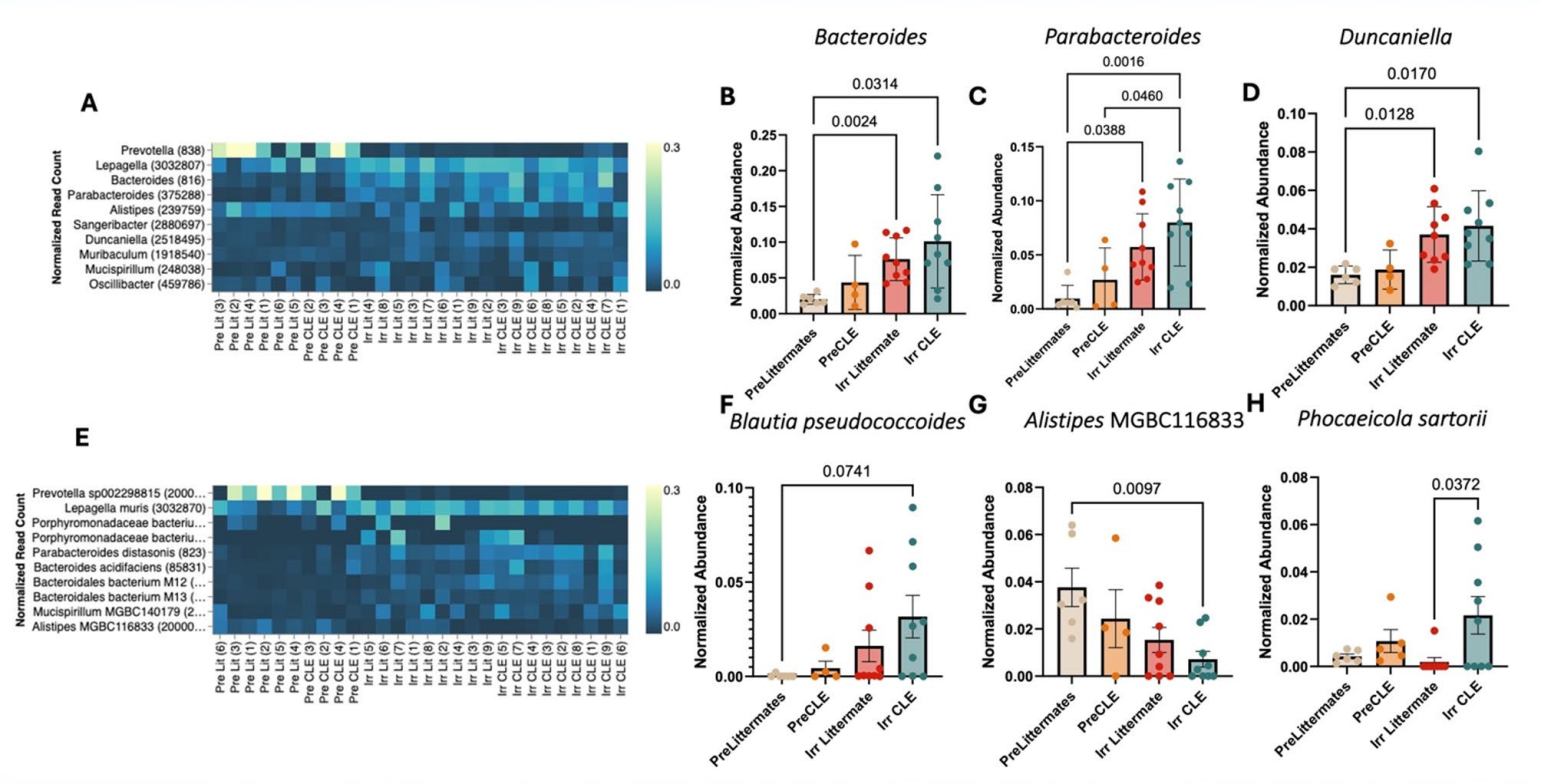
Examination of species and strains significantly increased in CLE mice compared to irradiated littermates. **A**. Heatmap of top 10 genera by disease group. **B**. *Bacteroides*, **C**. *Parabacteroides* and **D**. *Duncaniella* serve as examples of species that are enriched in post-induction CLE mice. **E.** Heatmap of top 10 strains by disease group. **F**. *Blautia pseudococcodies* is increased in post-induction CLE mice. **G**. *Alistipes MGBC116833* is decreased in post-induction CLE mice. **H**. Normalized abundance of *Phocaeicola sartorii* (*p* < 0.05) by disease group. (One-way ANOVAs with posttests significant as indicated; panel H demonstrates a t test for *P. sartorii* littermates versus CLE mice as it was identified in a larger correlation matrix screen and was thus specifically tested for differences across post-induction genotypes). Pre-induction mice pooled from *n* = 2 experiments, *n* = 4 pre-induction littermates and *n* = 6 pre-induction CLE mice. Post-induction littermates pooled from *n* = 3 experiments, *n* = 9 post induction littermates and *n* = 9 post-induction CLE mice.

We next asked whether we could identify specific strains that associate with disease status, with the goal of identifying potential treatment targets or probiotics. Examining the top 10 species by disease group identified stepwise increases in *Bacteroides*, *Parabacteroides* and *Duncaniella*, with the lowest abundance in pre-induction littermates and the highest abundance in post-induction CLE mice (Fig. 4A–D). Examining the top 10 strains by disease group identified a similar stepwise increase in *Blautia pseudococcoides*, and a stepwise decrease in *Alistipes MGBC116833* (Fig. 4E–G). *Phocaeicola sartorii* was one of the few strains that was significantly increased in post-induction CLE compared to littermate mice (Fig. 4H). Taken together, these data identify specific bacterial strains that might be useful targets for development of probiotics or strain-specific lysis such as through bacteriophage therapy.

### Specific strains correlate with immune infiltrates in skin as well as abundance in lymphoid organs

**Fig. 5:**
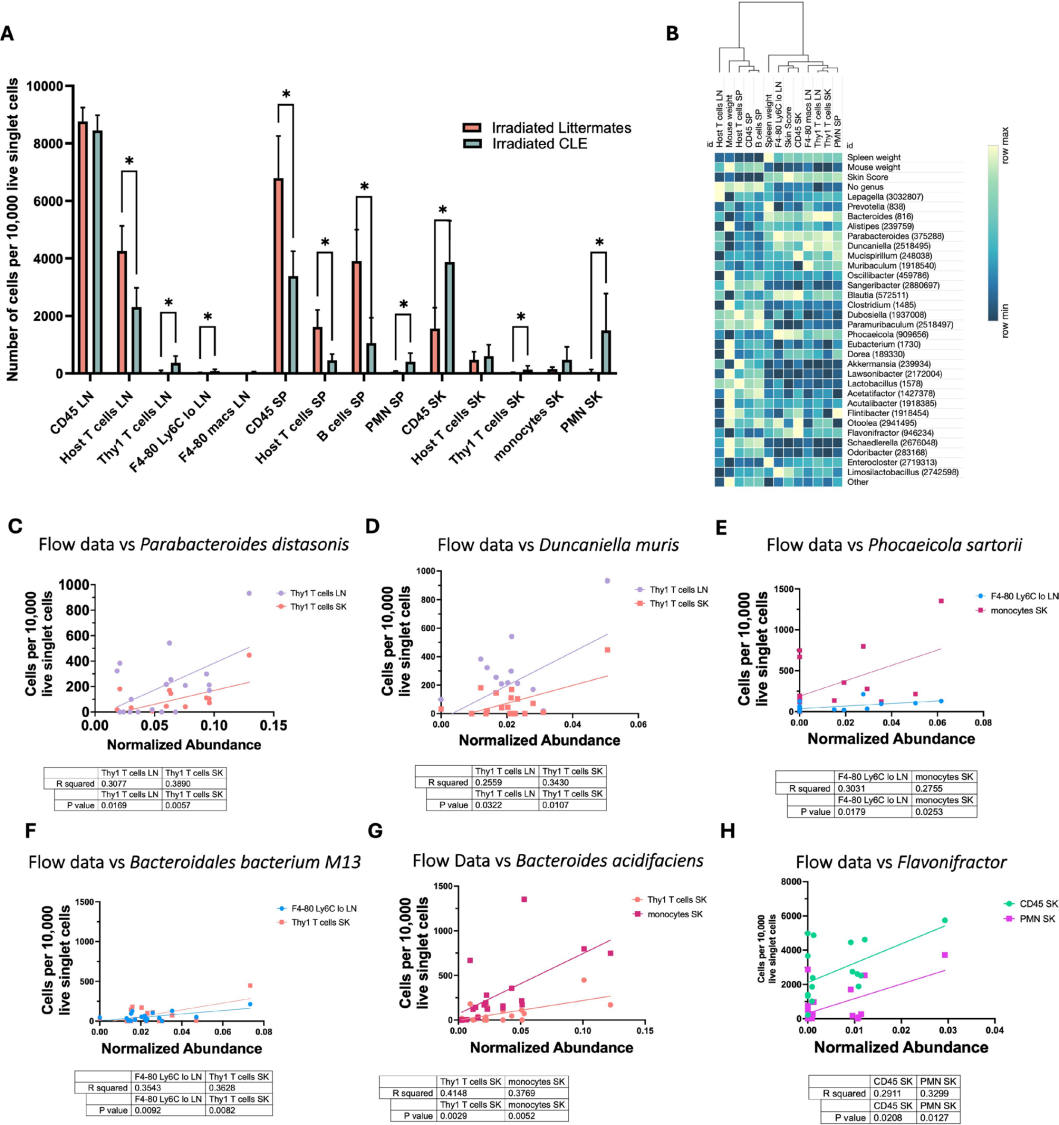
Immune data correlates with disease status, and specific species correlate with a higher abundance of disease-related immune cell subsets lymph nodes and skin. **A**. Quantified flow cytometry data by experimental irradiated groups. **B**. Heatmap of significant flow cytometric parameters versus spleen weight, mouse weight, and genera sorted by 1-pearson coefficient on columns. **C.** Thy1 T cells in lymph nodes and spleen versus *P. distasonis*. **D.** Thy1 cells in lymph nodes, skin versus *D. muris.* **E**. F4-80 Ly6C^lo^ cells in lymph nodes, monocytes in skin versus *P. sartorii.* **(F)** F4-80 Ly6C^lo^ cells in lymph nodes, Thy1 T cells in skin versus *B. bacterium M13.* **(G)** Thy1 T cells and monocytes in skin versus *B. acidifaciens.* **G.** CD45 and PMNs in skin versus *Flavonifractor*. Pearson R and P values significant as indicated, *n* = 9 irradiated littermates and *n* = 9 irradiated CLE mice pooled from *n* = 3 experiments.

To understand the potential impact the gut microbiome could have on skin disease in our lupus model, we examined immune data across disease cohorts and correlated these data with microbiome data. We gated immune cells in the lymph node (LN), spleen (SP) and skin (SK) in post-induction littermates and CLE mice to identify which populations were significantly different in diseased animals (Fig. 5A, Table S1). Next, we correlated these significantly different immune cell populations with the top microbiome genera, namely host T cells in the lymph node, mouse weight, host T cells in the spleen, CD45 cells in the spleen, B cells in the spleen, spleen weight (splenomegaly), F4-80 Ly6C^lo^ monocytes/macrophages in the lymph node, skin score, CD45 cells in the skin, F4-80 macrophages in the lymph node, and antigen specific T cells in the lymph node and skin (Fig. 5B). Heatmap matrix of bacteria genera and key immune data showed associations of higher *Bacteroides*,* Parabacteroides*,* Duncaniella* with higher skin scores, spleen weights, and specific cell lines related to disease, whereas *Akkermansia*,* Lawsonibacter*,* Lactobacillus*,* Schaedlerella*, and *Odoribacter* had negative associations with disease related flow data. Next, we examined strains that correlated with specific immune cell populations as identified in our correlation matrix, which we present in Fig. 5C-H. Specifically, we found positive correlations between *Parabacterodies distasonis* and *Duncaniella muris* with antigen-specific T cells in the LN and SK; *Phocaeicola sartorii* with F4-80 Ly6C^lo^ monocytes/macrophages in the LN and monocytes in the SK; *Bacteroidales bacterium M13* with antigen-specific T cells in the SK and F4-80 Ly6C^lo^ monocytes/macrophages in LN; *Bacteroides acidifaciens* with antigen-specific T cells and monocytes in SK; and *Flavonifractor* with total CD45 + and neutrophils (PMN) in SK.

**Fig. 6:**
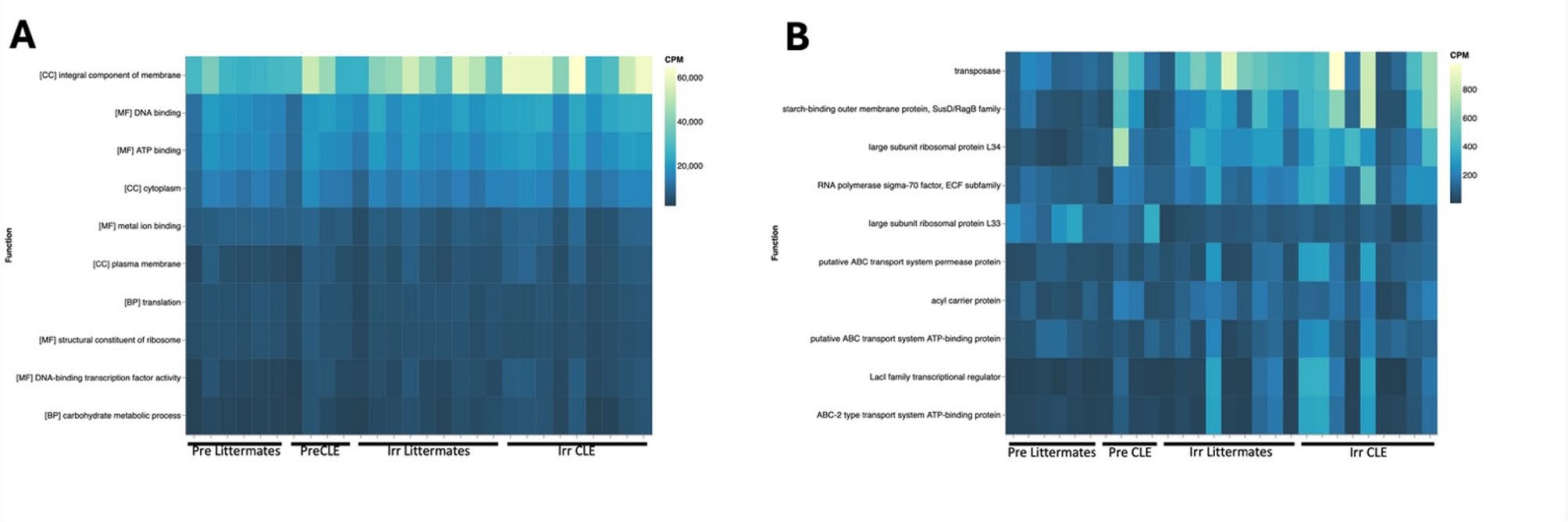
Functional microbiome data in gut samples shows enhancement of integral membrane protein functional pathways in CLE mice. **(A)** Top 10 GO Term functional parameters in gut samples in copies per million. **(B)** Top 10 KO Term^16–18^ functional parameters in gut samples in copies per million. Pre-induction mice pooled from *n* = 2 experiments, *n* = 4 pre-induction littermates and *n* = 6 pre-induction CLE mice. Post-induction littermates pooled from *n* = 3 experiments, *n* = 9 post induction littermates and *n* = 9 post-induction CLE mice.

We also performed functional gene analyses on the bacterial strains and noted an increase in the term “integral membrane protein functional pathways” in CLE post-induction mice (Fig. 6). We hypothesize that this may relate to immune cell activation, as synthesis of bacterial membrane proteins, including upregulation of receptors, transport and membrane binding components, contribute to immune cell activation and extravasation to skin.

### A preliminary analysis finds loss of specific fungal strains is associated with CLE disease

We also compared fungi abundance across cohorts, and expressed reads as percentages of overall number of classified reads for respective subjects. Fungi read counts were overall low in gut samples (< 100); therefore, the data is of limited internal validity and was insufficient to calculate abundance by OneCodex. Though these findings must be interpreted cautiously, overall trends showed lower proportions of *Colletotrichum tofieldiae* (*p* < 0.05) and *Verticillium* in CLE gut microbiome compared to littermates, and an increased proportions of *Periglandula ipomoeae* and *Penicillium paneum* in CLE compared to littermates, though these changes were similar to differences in pre-irradiation CLE mice versus littermates (Fig. S4). Taken together, these data demonstrate the effect that genotype and disease induction (irradiation, doxycycline administration, T cell injection) has on the gut mycobiome.

### Comparison of findings to publicly available studies of mouse and human lupus microbiomes

Several of our findings were compatible with existing literature in alternative lupus mouse models and human studies. These results are reported in Table 1. While a majority of these studies involve SLE patients or murine models more consistent with SLE, the overlapping results with our CLE model indicate the findings may be conserved across disease states and serves as further justification for use of this mouse model in future interventional studies.

**Table 1:** Comparison of existing literature of other lupus microbiome studies to this study.

Mouse studies						
Model type	Sample size	Findings in disease compared to control or pre-disease (Significance when indicated)			Notes	References
		No difference	Increased	Decreased		
NZB/W F1	n = 5 Pre-disease n = 3–4 Post-disease		↑ *Oscillospira* (p < 0.01) ↑ *Bilophilia* (p < 0.01) ↑ Runimoccaceae unnamed genus (p < 0.01) ↑ *Clostridium* (p < 0.01) ↑ *Dehalobacterium* (p < 0.01) ↑ *Lactobacillus* (p < 0.01)	↓ ***Akkermansia*** (p < 0.01) ↓ *Anaerostipes*	Human arm noted below In our study, *Akkermansia* negatively associated with disease related increases in flow data and had the biggest proportional negative change (**Sup **Fig. 3)	^8^
PIL BALB/cByJ	n = 5 PIL n = 5 Control	** ~ F/B ratio** ** ~ Alpha diversity**	↑ *Lactobacillus* ↑ Tannerellacea (family) ↑ ***Parabacteroides*** ↑ ***Bacteroides*** ↑ *Alistipes*	↓ Tenericutes (phylum)	Taxa were identified by concordance with human arm, noted below In our study, *Bacteroides, Parabacteroides* were positively associated with cell lines related to disease	^19^
PIL mice with FMT from either MRL/Lpr or MRL/Mpj mice	n = 11 PIL FMT-Lpr n = 10 PIL FMT-Mpj n = 7 PILFMT-PBS		↑ *Bilophila* (p < 0.05) ↑ *Streptococcus* (p < 0.05)	PIL-FMT(Mlp) compared with PIL- FMT(PBS): ↓ F/B ratio ↓ *Prevotella* (p < 0.05) ↓ *Alloprevotella* (p < 0.05)	If no specification, findings were consistent across both groups (FMT compared to PBS transplant and Mpj transplant)	^20^
MRL/Lpr	n = 6 MRL/lpr (Butyrate treatment group) n = 6 MRL/lpr (PBS treatment group) n = 8 BALB/c (control)		↑ Bacteroidota (phylum) ↑ Bacteroidales (order) ↑ Bacteroidia (class)	↓ Alpha diversity ↓ Bacillota (formerly Firmicutes) (phylum) ↓ Clostridia (class)	Comparison between pre-treatment MRL/Lpr mice compared to control BALB/c In our study, genus *Bacteroides* is increased, which is within the class Bacteroidia	^21^
MRL/Lpr	n = 3 MRL/Lpr n = 3 MRL/Mp		↑ Faith’s phylogenetic diversity ↑ Lachnospiraceae (family) (p < 0.05) ↑ Rikenellaceae (family) (contains *Alipstipes* genus) (p < 0.05) ↑ Streptococcaceae (family) (p < 0.05) ↑ Clostridiales family XIII (p < 0.05)	↓ Lactobacillaceae (family) (p < 0.05)		^22^
MRL/Lpr	n = 7 MRL/Lpr (PBS treatment) n = 7 MRL/Lpr (*Lactobacillus* treatment) n = 4 MRL/Lpr mice n = 4 MRL mice			↓ Lactobacillales (order)	Weekly gavages of *Lactobacillus* spp. significantly reduced autoantibodies in the circulation (p <0.05) and proteinuria (p < 0.05) compared to PBS gavage	^23^
*Nfkbiz* ^ΔK5^	n = 3 Nfkbiz^f/f^ n = 3 Nfkbiz^ΔK5^		↑ *Staphylococcus aureus* colonization of skin (p < 0.05)			^13^

Human studies						
Disease	Sample size	Findings in disease compared to control or pre-disease (Significance when indicated)			Notes	References
		No difference	Increased	Decreased		
SLE	n = 20 SLE n = 20 healthy controls			↓ F/B ratio ↓ *Lactobacillus* (p < 0.006)		^10^
SLE	n = 16 SLE n = 76 matched healthy controls		↑ Tannerellaceae (family) (p < 0.01) ↑ *Alistipes* (p < 0.05) ↑ ***Flintibacter*** (p < 0.05) ↑ ***Parabacteroides*** (p < 0.01) ↑ *Bacteroides thetaiotaomicron* (active SLE subgroup) ↑ *Ruminococcus gnavus* (active SLE subgroup)	↓ F/B ratio (p < 0.05) ↓ Alpha diversity (p < 0.01) ↓ Lactobacillaceae (family) (active SLE subgroup)	Our study also found modest but insignificant proportional increases in Flintibacter in CLE mice compared to post-induction littermates (**Sup **Fig. 3)	^19^
SLE	n = 14 SLE N = 17 non-SLE controls	** ~ F/B ratio**	↑ Proteobacteria (phylum) (p < 0.05) ↑ ***Blautia* species** ** (p < 0.05)**	↓ Rikenellaceae (family) genus (p < 0.05) ↓***Odoribacter* species** (p < 0.05)	Our study found modest but insignificant proportionate increases in *Blautia*,and decreases in *Odoribacter* (**Sup **Fig. 3)	^8^
SLE	n = 21 SLE n = 10 healthy controls	** ~ Alpha diversity**		↓ Bacteroidota (phylum) ↓ Clostridia (class) ↓ Ruminococcaceae (family)	KEGG pathway analysis indicated increases in lipid and amino acid pathways in SLE patients compared to healthy	^24^
SLE (CLE lesions)	n = 54 SLE n = 6 psoriasis		↑ *Staphylococcus aureus* (p < 0.0001)		This finding was a comparison of 14 SLE rash swabs compared to 6 psoriasis rash swabs	^25^
CLE	n = 6 CLE (petroleum treatment group) n = 6 CLE (2% mupirocin treatment group)				Proof of concept of above study, patients were treated with Mupirocin and decreased *Staphylococcus aureus* colonization of lesions was correlated with decreased IFN pathway signaling and inflammatory gene dysfunction	^26^

Text is bolded if findings are similar to findings reported in this study. ↑ indicates increased, ↓ decreased, ~ indicates no difference was found. PIL = pristane-induced lupus, FMT = fecal microbiome transplant, PBS = phosphate buffered saline, SLE = systemic lupus erythematous, SCLE = subacute cutaneous lupus erythematous, IFN = interferon.

## Discussion

Current first line treatment for SLE or CLE is largely nonspecific immune suppression, which increases risk of infections and malignancy, among other short- and long-term side effects. While changes in the microbiome have been investigated in both murine models and humans with SLE, limited research has focused on identifying specific microbes that may potentiate disease or serve as candidates for probiotic-based intervention. Lupus mouse models and more recent human studies support a promising role for dietary interventions that increase microbial diversity and decrease inflammation^6^. Thus, characterization of the microbiome and mycobiome can set the groundwork for further mechanistic and interventional studies.

We found no significant differences in F/B ratios from our mice, though normalized read counts of the top 10 phyla demonstrated an increase in Bacillota (formerly Firmicutes) and a decrease in Bacteroidota in CLE mice post-induction. Interestingly, these findings are consistent with other SLE murine models in which there were no significant differences in F/B ratios between diseased and control mice, despite there being notable changes in the gut microbiome associated with disease progression and remission (Table 1)^27^. Literature suggests that F/B ratios are lower in mice than in humans and caution is needed when generalizing F/B ratios in model organisms as they may not reliably recapitulate the human gut microbiome^28^. Though alpha diversity trended lower, beta diversity by Simpson index was similar across cohorts. Notably, Shannon beta diversity, which gives more weight to rare species, was more different between CLE mice and littermates, indicating greater reductions in more rare species in diseased mice.

The model induction process, which involves administration of doxycycline chow to turn on the model autoantigen and 400R irradiation to make room for antigen-specific T cells, impacts the microbiome in both CLE mice and littermate controls. Specifically, we noted a significant loss of *Prevotella*, which is also observed to be lost in human gut microbiomes in response to Western diet^29^. *Prevotella copri* was recently reported to be lost in Korean SLE patient gut microbiomes^30^. *Prevotella* is sensitive to doxycycline, though it is unclear whether the dose administered to the mice (20 mg/kg ad libitum) was responsible for the observed loss as long-term doxycycline administration at 20 mg sub-antimicrobial doses has been reported to not have significant effects on gut or vaginal microbiota in humans^31^. Low dose irradiation was reported to reduce alpha diversity in mice, as well as specific metabolites^32^. It would be interesting to study whether the specific loss of *Prevotella* and reduced alpha diversity is sufficient to allow for the development of cutaneous lupus in mice as a result of the disease induction protocol.

As doxycycline chow is commonly used to activate disease states in transgenic mouse models, previous work has investigated changes in the microbiome after doxycycline treatment. For example, in CaMK2α-tTA mice, a model for neuroinflammatory conditions, alpha diversity was notably reduced after dox induction in both TG and WT mice, and this change persisted after doxycycline washout period^33^. We also observed reduced alpha diversity measures in both littermates and CLE mice after induction (Fig. 3C). Similarly, C57BL/6NCrl mice who received doxycycline chow had distinct microbiome composition compared to mice who did not receive doxycycline^34^. In honeybee models, decreases in *Bifidobacterium* and *Lactobacillus* were identified with tetracycline treatment^35^. Likewise, irradiation in mice significantly reduced the genus *Lactobacillus*, however, did not impact the overall alpha diversity nor the overall abundance of *Bacillota* or *Bacteroidota* phyla^36^. It would be plausible that the lack of *Lactobacillus* findings in our model may have been a result of the induction protocol, given that several SLE mouse models have found enrichment of *Lactobacillus* strains in disease, and alterations in disease status based on *Lactobacillus* supplementation. Conflicting results of probiotic supplementation of in SLE mouse models and in human studies make *Lactobacillus* species’ role unclear^37^.

We found significant increases in *Bacteroides*,* Parabaceteroides*,* Duncaniella*,* Blautia pseudococcoides* and *Phocaeicola sartorii* in our post-induction CLE mice compared to other groups, indicating that these strains might be associated with lupus inflammation. *Bacteroides* and *Parabactererodies* species are increased in human lupus gut microbiomes, specifically in glucocorticoid negative patients^38^. This fits with our mouse model, as we did not provide glucocorticoids to the mice. *Duncaniella* was increased in a study that performed fecal transplantation through feeding of WT or control (non-diseased) mouse fecal pellets to SLE-prone mice^27^. It is possible that our mice had increases in *Duncaniella* through coprophagy from littermates that were housed together in their cages. However, it is unclear why they would have higher levels compared to littermates, unless there is an effect of genotype and/or inflammatory status on abundance of this phylum. A recent study characterizing microbiome changes in lupus patients identified *Blautia gnavus* blooms during flares^39^. *B. gnavus* used to be considered a part of the *B. pseudococcoides* family but was recently reclassified^40^. It would be interesting to further investigate whether or not these strains perform homologous functions in the context of lupus across species. *P. sartorii* was recently reported to be enriched in the gut of mild, but not severe, lupus in MRL/lpr mice^41^. Given that our model primarily impacts the skin, with mild if any kidney disease, this fits with this observation. Other *Phocaeicola* strains have been suggested to alleviate inflammation, such as *P. dorei* and *P. vulgatus*, which were shown to regulate pro-inflammatory cytokines and produce beneficial short chain fatty acids^42^. However, it is notable that particular strains of these species may cause benefit while other strains may have the opposite effect. This finding in which different species of the same genus, or even strains within the same species, confer benefit while others are harmful is similar to the disparate findings of different *Lactobacillus* species, and warrants further investigation^37^.

Antigen-specific T cell infiltration into the skin was positively correlated with *Parabacteroides distasonis*,* Duncaniella muris*,* Bacterodiales bacterium M13* and *Bacteroides acidifaciens*. *P. distasonis* was recently reported to promote CXCL9 secretion by tumor-associated macrophages which in turn promoted CD8 + T cell activation and anti-tumor immunity in the context of lung cancer^43^. How this activity could influence skin infiltration of T cells in an autoimmune setting requires further investigation. *D. muris* was recently described to have different clinical isolates^44^, one of which is associated with anti-inflammatory properties in a DSS colitis model^45^. It is unclear whether the isolate in our mice is pro- or anti-inflammatory, though our data suggests that it might act in a pro-inflammatory capacity in the TLR9KO genetic background, given the increase in antigen-specific T cells in the skin. Antigen presenting cell populations’ infiltration into the skin, including monocytes (Ly6C + Ly6G-) and F4-80 + Ly6C^lo^ macrophages, were positively correlated with *P. sartorii* and *B. acidifaciens*, with higher lymph node numbers positively correlated with *P. sartorii* and *B. bacterium M13*. How *P. sartorii* or *B. bacterium M13* impact monocyte and macrophage populations remain unclear. *B. acidifaciens* is associated with inflammation-induced tumorigenesis in DSS models, though the effect it might exert on tumor-associated macrophages is unclear^46^. Last, neutrophils and total CD45 + inflammatory cells were positively correlated with *Flavonifractor*. *Flavonifractor* is increased in the gut of bullous pemphigoid patients, providing a gut-skin connection with relevance to a skin disease that is also characterized by neutrophilic infiltration^47^. It would be interesting to test targeting of each of these strains in CLE to determine the impact on skin inflammation and clinical disease scores. This could be achieved through bacteriophage therapy, which is currently being investigated primarily for infectious diseases^48^. One specific example of this is a preclinical phage therapy that improved immunity to *S. aureus* in immunocompromised mice^49^. Given that *S. aureus* is enriched in CLE skin^12^, it could be a promising hypothesis to test whether phage therapy may have therapeutic benefit for lupus skin disease,

Microbiota have been investigated to impact metabolism through augmentation of human metabolic pathways, a well-known symbiotic relationship in which bacteria supply enzymatic capabilities its host would otherwise not have^50^. Functional data in our model showed an upregulation of membrane proteins, which includes transporter families and cellular attachment and signaling proteins such as lectins and binding receptors. All of these ligands have the potential to stimulate innate immune receptors, which could contribute to lupus immunopathogenesis^51^. While our model does not show differences in short chain fatty acid pathways in the functional results, murine studies have explored the impact of butyrate supplementation on SLE, as butyrate is thought to be a beneficial byproduct of bacterial fermentation^21^. Interestingly, doxycycline and tetracycline treatments are thought to reduce butryate-producing bacteria in other disease such as acne, and therefore theoretically could have been impacted by our model induction process^52^.

Studies have also demonstrated changes in the gut’s fungal populations, or the mycobiome, in SLE^53^. One pilot study found human SLE mycobiomes demonstrated distinct dysbiosis compared to healthy controls and rheumatoid arthritis^54^. Another study reported different mycobiome beta diversities in three cohorts of SLE with lupus nephritis (LN), SLE without LN, and healthy controls, with both SLE cohorts having increased ratio of opportunistic fungi and *Aspergillus* being correlated with 24 h proteinuria, anti-dsDNA and ANA^55^. Murine lupus models including FcGRIIb deficient mice and pristane treated mice exhibit an elevated Basidiomycota-to-Ascomycota ratio that was positively correlated with disease severity^22^. In our model, the fungal strain *Penicillium paneum* was higher in CLE mice versus littermates, while *Colletotrichum tofieldiae* was absent. Low fungal read counts severely limit interpretation of this data, and therefore further study would be needed to determine if these findings could be replicated. We present this as a preliminary analysis, and hypothesize that fungal probiotics that are lacking in CLE post-induction mice could be further tested for utility as probiotic strains. *Colletotrichum tofieldiae* is a particularly interesting candidate given it is considered a beneficial root probiotic^56^, and might be enriched in whole food diets^57^.

Limitations of our study include small sample size which limited power to detect significant differences. The sample size allocated for this study was prohibited by cost, space, and time, but based on previous mouse studies outlined in Table 1, was deemed to be reasonable for investigation. Mice were selected from several experiments that occurred at separate times, in a clean but not germ-free environment. Intermouse grooming and coprophagia may introduce microbiome congruence across disease cohorts. The mouse model itself relied on irradiation for induction, which impacts the microbiome significantly. For this reason, the irradiated littermate cohort was used as a comparison group to mitigate the confounding impact of irradiation on the microbiome. Males and females were analyzed together based on our PCA analysis, though sex hormones have been shown to play a role in microbiome composition in other murine models. While analysis of the gut microbiome in murine models is useful for hypothesis generation and particularly for interventional studies involving the microbiome, the mouse microbiome cannot be directly extrapolated to humans. Post hoc comparisons were made after identification with multivariable regression in this model with raw p values are reported; these results must be interpreted with caution as further testing is needed with interventional studies for biologic significance and impacts on downstream signaling pathways. Last, this study demonstrates associations which we may compare to other mouse models and human changes, but further experimentation such as fecal microbiome transplantation would be ideal to establish whether these microbes cause disease, mitigate disease, or are a consequence of disease induction. Characterization of notable changes as outlined in this paper will establish a foundation for future experimentation in this CLE mouse model, for which no understanding currently exists.

In conclusion, we present this paper as a characterization of a CLE mouse model’s gut microbiome for future interventional study, as well as for hypothesis generation in further study in humans. Future directions include investigation of the skin microbiome in this model, as well as mechanistic studies understanding how specific strains and populations impact disease progression are planned.

## Materials and methods

### Mouse model and fecal pellet sampling

Animal protocol was reviewed and approved by the UMass Chan IACUC (#202100229), and studies were conducted in accordance with the National Institutes of Health (NIH) Guide for the Care and Use of Laboratory Animals. Eighteen (18) mice were selected for analysis from a representative cohort of CLE mice and littermate controls from 2 to 3 separate experiments. Disease was induced as previously described^14,15^ using 10 million Th2 skewed OT2 CD4 + T cells adoptively transferred into sublethally irradiated recipient mice (400R). Mice also received 200 mg/kg doxycycline chow (BioServ) ad libitium to turn on the ovalbumin model autoantigen in keratin 5 expressing cells (keratinocytes). Both littermates and CLE mice received irradiation and doxycycline chow. Littermates, who lacked localized keratinocyte Ova expression, were either injected or not injected with Th2 cells for different sets of negative controls. Model diagram is depicted in Fig. 1. Severity of skin lesions were collected weekly on a 0–4 scale, with 0 indicating no skin changes and 4 indicating at least 75% skin involvement. Stool samples from pre- (week 0) and post-induction (week 3–4) were snap frozen at -70 C and batched for analysis. We confirm the study design is reported in accordance with ARRIVE guidelines.

### Microbiome sample preparation

Transnetyx Microbiome kits containing barcoded sample collection tubes were provided by Transnetyx (Cordova, TN, USA). Mouse fecal samples were placed in individual tubes containing DNA stabilization buffer to ensure reproducibility, stability, and traceability, and shipped for DNA extraction, library preparation, and sequencing by Transnetyx (Cordova, TN USA).

### DNA extraction and metagenomic sequencing

DNA extraction was optimized and fully automated using a robust process for reproducible extraction of inhibitor-free, high molecular weight genomic DNA that captures the true microbial diversity of stool samples. After DNA extraction and quality control (QC), genomic DNA was converted into sequencing libraries using a method optimized for minimal bias. Unique dual indexed (UDI) adapters were used to ensure that reads and/or organisms are not mis-assigned. After QC, the libraries were sequenced using the shotgun sequencing method (a depth of 2 million 2 × 150 bp read pairs), which enables species and strain level taxonomic resolution. Sequencing data were uploaded automatically onto One Codex analysis software and aligned against the One Codex database consisting of ~ 148 K complete microbial genomes, including 71 K distinct bacterial genomes, 72 K viral genomes, and thousands of archaeal and eukaryotic genomes. The classification results were filtered through several statistical post-processing steps designed to eliminate false positive results caused by contamination or sequencing artifacts. Samples were compared with TransnetYX’s global diversity averages from historic data for quality control check. Low readcounts were proportionalized to the total number of identifiable reads from their host sample. Sequencing data was aligned with the Gene Ontology (GO) and KEGG Orthology databases^16–18^ for functional analysis on the OneCodex platform.

### Statistical analysis

OneCodex python package, Jupiter notebooks, and GraphPad Prism were used for analysis. All data are displayed as mean ± SD. For identification of significant immune cell populations and differential expressed bacterial taxa, multiple t-testing between post-irradiation littermates and CLE mice was used with FDR correction to control for multiple tests. Differentially expressed immune cells were discovered with this testing, however no bacterial taxa were significant, likely due to a higher number of tests between taxa that made discovery difficult with multiple testing corrections. Therefore, multivariate analyses of skin score versus relative abundance of bacteria were repeated at different taxonomic levels using GraphPad Prism. Taxa with significant p-value correlation (*p* < 0.05) to skin score were extracted to compare across cohorts. We accepted raw p values for further analysis because prevailing theories for microbiome-immune system interaction include direct immune stimulation, disruption of barrier integrity, or active metabolite and cytokine production, in which immune signaling cascades produce exponentially larger biologic effects than downstream of raw abundance. Thus, we felt that at our analysis level upstream of signal cascades, differences may be smaller than expected but maintain large biologic effects which require further investigation. Gaussian distribution was verified using the Anderson-Darling test. One-way ANOVAs with posttests were performed to compare all 4 groups (littermates pre-induction, littermates post-induction, CLE pre-induction, CLE post-induction), and normality tests and tests for standard deviation variations were conducted to ensure the correct type of ANOVAs and post-tests were selected. We also employed an unpaired t-test with Welch’s correction to compare between littermates and CLE mice post-disease induction when data were normally distributed. Mann-Whitney test was used when data were non-normally distributed. Network analyses are constructed by OneCodex package on Jupytr and Morpheus.

## Supplementary Information

Below is the link to the electronic supplementary material.

Supplementary Material 1

Supplementary Material 2

Supplementary Material 3

Supplementary Material 4

## Data Availability

The datasets generated and/or analysed during the current study are available in the OneCodex repository, available via the following links: Gut microbiome project: [https://app.onecodex.com/projects/120f7b4173934b33](https:/app.onecodex.com/projects/120f7b4173934b33) Unifrac and heatmaps Jupytr notebook: [https://app.onecodex.com/notebooks/public/02ad540f05e6424a](https:/app.onecodex.com/notebooks/public/02ad540f05e6424a) PCAs and alpha/beta diversity Jupytr notebook: [https://app.onecodex.com/notebooks/public/fca60f7f41ca4dab](https:/app.onecodex.com/notebooks/public/fca60f7f41ca4dab) A static copy of the code is also provided in the supplementary materials.
