# Supplementary material for: Gut dysbiosis in a murine model of cutaneous lupus erythematosus correlates with antigen-specific T cells and antigen-presenting cells in skin
Source: Sci Rep. 2026 Jan 12;16:4511. doi: 10.1038/s41598-025-34741-6 (PMC12864916; doi:10.1038/s41598-025-34741-6)
Supplement: Supplementary file 2 — Supplementary Material 2 [file 41598_2025_34741_MOESM2_ESM.docx]

**Gut Dysbiosis in a Murine Model of Cutaneous Lupus Erythematosus Correlates with Infiltration of Antigen-Specific T cells and Antigen Presenting Cells in Skin**

Haley Neff1, Ümmügülsüm Yıldız-Altay1, Nuha Salam1, Doyle V. Ward2, Dominique Shepard3, Zaida G Ramirez-Ortiz3, Jillian M Richmond1,4*

**Supplemental Figures & Legends**:


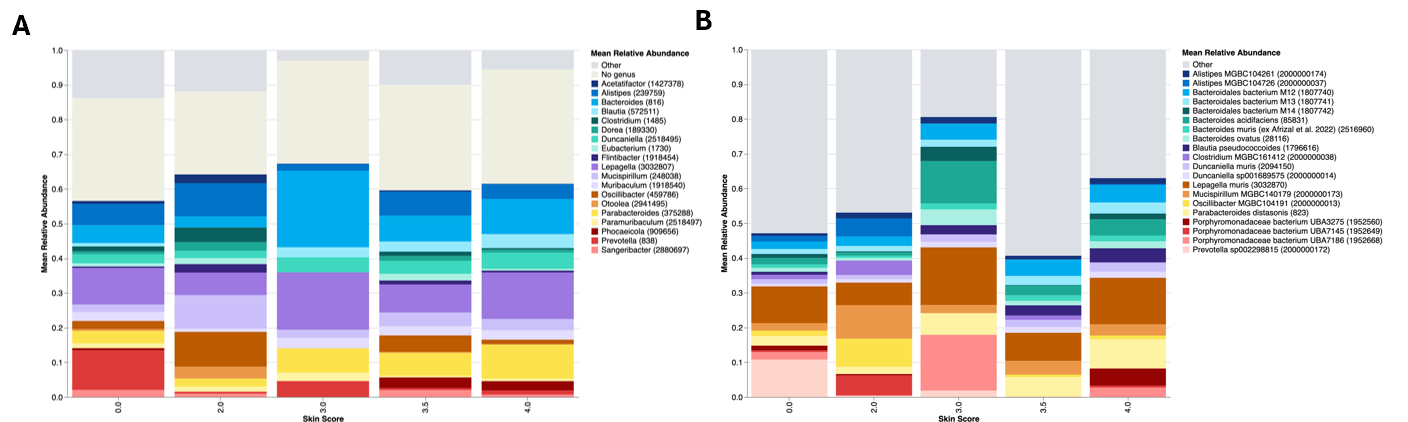


**Figure S1. Relative abundance plots by skin score. A.** Relative abundance plot of top 20 genera and **B.** top 10 species binned by skin score (n= 19 score 0, n = 1 score 2, n = 1 score 3.0, n = 2 score 3.5, and n = 5 score 4 mice).


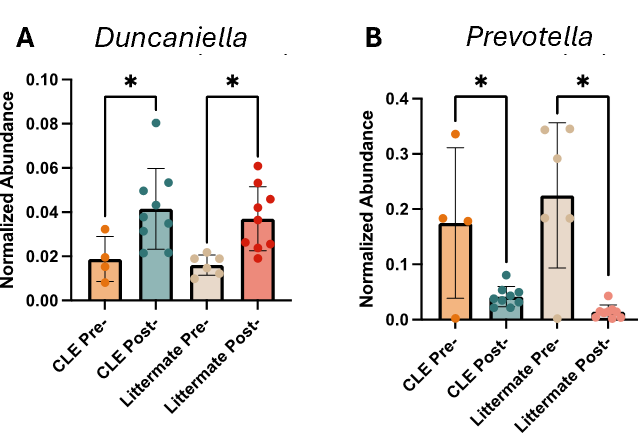


**Figure S2. Relative abundance of some genera increased after irradiation, while others decreased. A**. Abundance of genus *Duncaniella* pre- and post-irradiation by experimental group. **B.** Abundance of genus *Prevotella* pre- and post-irradiation by experimental group. * indicates p < 0.05.


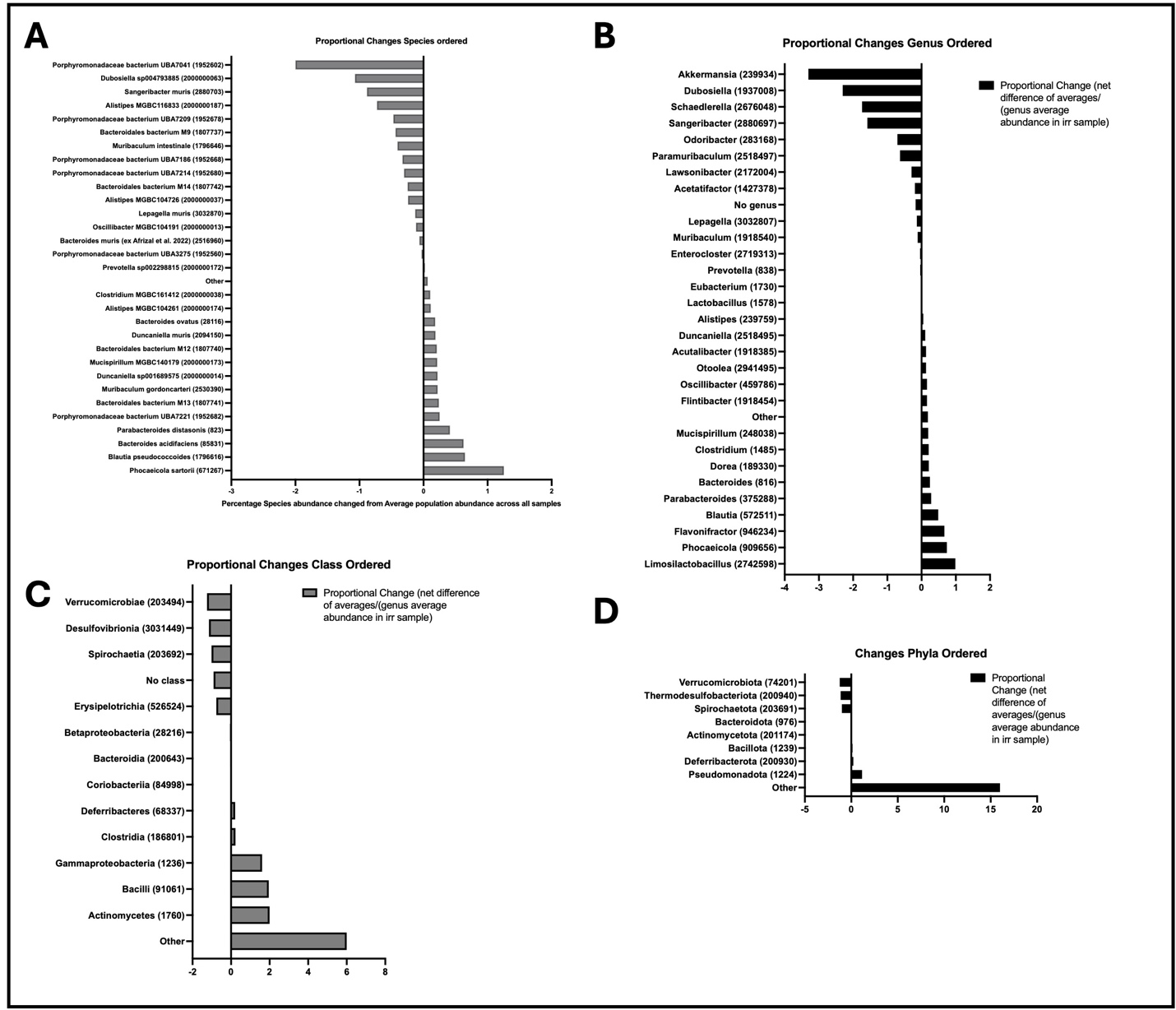


**Figure S3. Proportional changes in abundance of specific taxa** (**A.** species, **B.** genus, **C.** class, **D.** phyla) in irradiated CLE compared to irradiated littermate mice.


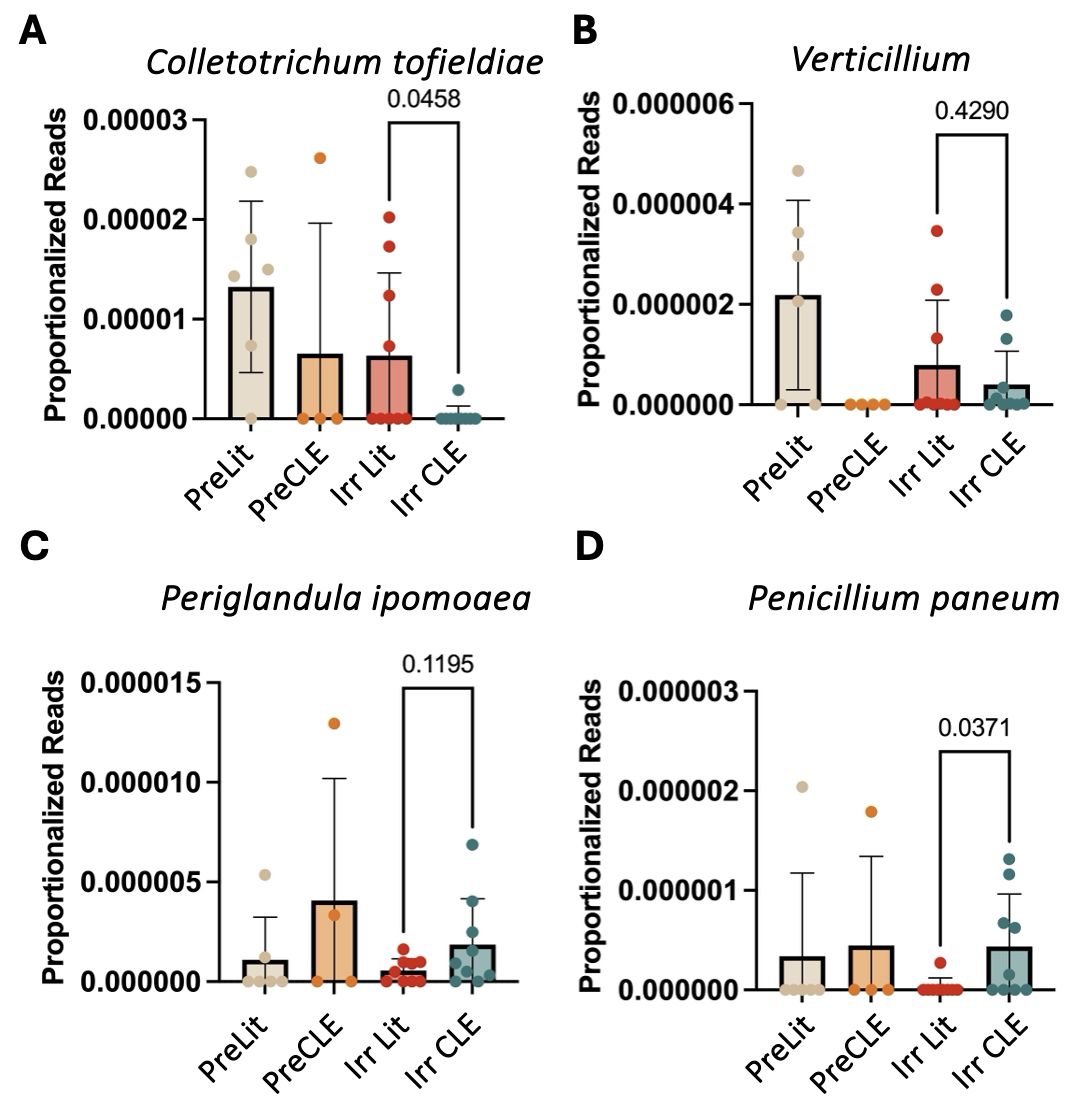


**Figure S4. Proportional abundance of specific gut mycobiome strains differ by disease group. A.** *Colletotrichum tofieldiae* by experimental group. **B.** *Verticillium* by experimental group**. C.** *Periglandula ipomoeae* by experimental group**. D.** *Penicillium paneum* by experimental group. Pre-induction mice pooled from n= 2 experiments, n = 4 pre-induction littermates and n = 6 pre-induction CLE mice. Post-induction littermates pooled from n = 3 experiments, n = 9 post induction littermates and n = 9 post-induction CLE mice.
