# Supplementary material for: Gut dysbiosis in a murine model of cutaneous lupus erythematosus correlates with antigen-specific T cells and antigen-presenting cells in skin
Source: Sci Rep. 2026 Jan 12;16:4511. doi: 10.1038/s41598-025-34741-6 (PMC12864916; doi:10.1038/s41598-025-34741-6)
Supplement: Supplementary file 3 — Supplementary Material 3 [file 41598_2025_34741_MOESM3_ESM.pdf]

One Codex Notebooks – Advanced Tutorial

1. Create a One Codex API connection

This first step connects your notebook to the One Codex API, providing secure access to your private data as well as publicly shared samples available on our platform.

In [1]:
```
from onecodex import Api

ocx = Api()
```

2. Fetch samples

Link to our onecodex project: https://app.onecodex.com/projects/120f7b4173934b33

In [2]:
```
# Fetch a project by its ID.
#
# You can also fetch your own projects by the `name` field using a `.where()`
# query. For example:
#
ocx.Projects.where(name='Gut microbiome')
#
# Alternatively, you can fetch a public project using its short `project_name`
# and by passing `public=True`. For example:
#
#ocx.Projects.where(public=True, project_name='Gut Microbiome')
#

project = ocx.Projects.get('120f7b4173934b33')
```

In [3]:
```
project
```

Out[3]:

	Projects('120f7b4173934b33')
description	''
external_id	None
name	'Gut microbiome'
owner	<Users 6d378ab2536149d7>
permissions	['can_add_files', 'can_administer', 'can_download_files', 'can_edit_metadata', 'can_incur_charges', 'can_see_files']
project_name	None
public	True

Next, let's fetch all the samples associated with the project

In [4]:
```
# This returns nothing because by default we search our *own* samples only
ocx.Samples.where(project=project)
```

Out[4]:
```
[<Samples b4f2a64b8ea540bb: "FS38906117.fastq.gz">, <Samples fa97ff9f2613408d: "F
S38906254.fastq.gz">, <Samples 0d717ee658504d64: "FS38993450.fastq.gz">, <Samples
0f63f4eb6b824530: "FS38994104.fastq.gz">, <Samples 874241b8d6774308: "FS68661606.
fastq.gz">, <Samples 88d3301cb5c94c10: "FS68661649.fastq.gz">, <Samples e1d4e0f42
9ea48da: "FS68661772.fastq.gz">, <Samples 4a3ace12e4eb4933: "FS68661818.fastq.g
z">, <Samples f9e560be678b4672: "FS68662119.fastq.gz">, <Samples e09fdf89876e416
f: "FS68662229.fastq.gz">, <Samples 007de7018e564b0e: "FS68662318.fastq.gz">, <Sa
mples b966ab22fc02460d: "FS68662320.fastq.gz">, <Samples 3c7b5fe6cee24add: "FS686
62393.fastq.gz">, <Samples 01c115ace07c41be: "FS68662593.fastq.gz">, <Samples 7c7
c4590b13b4032: "FS68662666.fastq.gz">, <Samples f99e271bd1c74179: "FS68662793.fas
tq.gz">, <Samples eef6a5c0c6654e2c: "FS68669789.fastq.gz">, <Samples 9043dcaa0b36
4510: "FS68670071.fastq.gz">, <Samples 790e8999dd2c4ef2: "FS68670134.fastq.gz">,
<Samples 552f1847d539483f: "FS68670142.fastq.gz">, <Samples 1343388a7512471c: "FS
38992039.fastq.gz">, <Samples 80123f524edd48ea: "FS38994645.fastq.gz">, <Samples
c2b7514503f8405a: "FS38994792.fastq.gz">, <Samples 7235873cf8424846: "FS38995936.
fastq.gz">, <Samples 7f2d0c253ca644a8: "FS38996041.fastq.gz">, <Samples e5fff06dd
f9948f0: "FS38996156.fastq.gz">, <Samples a342d2287fe4438f: "FS38996183.fastq.g
z">, <Samples 76376a4abda74fe1: "FS68624231.fastq.gz">]
```

In [5]:
```
samples = ocx.Samples.where(project=project, limit=50)
```

In [6]:
```
help(len)
```

```
Help on built-in function len in module builtins:

len(obj, /)
    Return the number of items in a container.
```

In [7]:
```
len(samples)
```

Out[7]:
```
28
```

Now, let's look at some of the samples associated with this study.

Note: These samples have a lot of custom metadata. You can add custom metadata to your samples on our website, by uploading an Excel sample sheet, or via our API. For more details, see our documentation on metadata: https://docs.onecodex.com/docs/metadata.

In [8]:
```
samples[0].metadata
```

Out[8]:

	Metadata('cc1a4a41b49e4dcd')
custom	{'Behavior testing': '170+ sec', 'Behavioral testing: Acceleration time test after 10 seconds': 170, 'Brain weight': 'NR', 'Cage': 94, 'Cells Injected': 'Noninjection\xa0', 'Cohort': 'After Irradiation', 'Days': 90, 'Disease': 'Irr_noninj', 'Donor DOB': 'N/A (uninj)', 'Donor age': 'N/A (uninj)', 'Experiment': 11, 'Experiment type': 'Flare TD', 'Experiment.1': 11, 'Expt start date': '1/12/2023', 'Genotype status': 'Littermate', 'IV or IP': 'None', 'Inject/non': 'Irr_noninj', 'Mouse ID': '94L', 'Mouse ID.1': '94L', 'Mouse age': 23, 'Mouse birth date': '8/3/2022', 'Mouse weight': 22.3, 'Name.1': '94L irradiated', 'Order 1': 11, 'Order Number': 33516, 'Post Groups': 'Uninj_lit', 'Pre/Post Groups': 'PostLit', 'Sample Origin': 'Fecal', 'Score': 0.0, 'Score.1': 0.0, 'Sex': 'F', 'Skin Score': 0.0, 'Sort order': 3, 'Speed-rpm': 20, 'Spleen Weight': 76.3, 'Spleen weight': 76.3, 'Status': 'Littermate', 'Status 1': 'Irr_Lit', 'Status2': 'Irr Lit', 'T cell status': 'Uninjected', 'TRE-2 Tg': '+', 'Tlr9-1 KO': '+', 'Tlr9-1 WT': '-', 'Treatment status': 'Untreated', 'Weight': 22.3, 'tRFP': '+', 'tTA': '-'}
date_collected	None
date_sequenced	None
description	('CLE-10 mice before and 3 weeks after irradiation\n' 'CLE-11 TRM mice before and 3 months after irradiation')
external_sample_id	'FS38906117'

	Metadata('cc1a4a41b49e4dcd')
library_type	None
location_lat	None
location_lon	None
location_string	''
name	'94L irradiated'
platform	'Illumina NovaSeq'
sample_type	None
sample	Samples('b4f2a64b8ea540bb')
starred	False
updated_at	datetime.datetime(2025, 6, 8, 14, 30, 47, 749682, tzinfo=TzInfo(0))

In [9]:

```
# To keep things speedy, we're going to work with a subset of the samples from th
# ten_samples = samples[:10]
```

3. Alpha diversity statistics

The `onecodex` Python client library provides rich access to the API layer of the One Codex platform. This includes several built-in functions for computing common alpha and beta diversity statistics. More statistics can be calculated by passing data from One Codex to the `skbio` library (the basis for much of QIIME2).

First, we'll get started by computing Simpson's index for our subset of ten samples. Below, `classification_id` is a unique identifier associated with each sample at One Codex.

In [10]:

```
samples.alpha_diversity('simpson')
```

Out[10]:

	simpson
**classification_id**	
**99bce79f5f9e46c4**	0.965950
**e95a62fa8fa8421c**	0.909253
**1b5fb828156a45f1**	0.948343
**fb7a978eba07450b**	0.863889
**c1c3306bcaa242f4**	0.969017
**1d4a86f3b8de4419**	0.966254
**3b4230c2315e4b38**	0.901315
**b0b0eed901884e5c**	0.933037
**8eb32381cb5847cc**	0.899714
**63a194f3415e4040**	0.956468
**f2f980f0d74d48b1**	0.944983
**e3f9c8b57d624c2f**	0.870377
**141ae8562eac4d74**	0.875178
**4aaae28c3bb14949**	0.959573
**6a835d31633948ad**	0.916227
**732fa96a0a334f4f**	0.904347
**c338cd9070c84ac4**	0.933352
**65536da7db814d0e**	0.952292
**c04e8bb1ab814c56**	0.963941
**d67f23f904634686**	0.958628
**bf8040ed1e744f4f**	0.938489
**2fefd1c1040a4870**	0.903883
**19f9bf1124584bba**	0.962307
**64ce2d6507f24d36**	0.916572
**47916150098d4399**	0.938825
**5e474f22ddf44548**	0.932590
**6674e09cac224504**	0.927172
**d49be622f7c4440c**	0.923799

Several other alpha (within-sample) diversity statistics are also included, e.g., the Chao 1 richness estimator. The results are returned as a pandas DataFrame, which can be manipulated to include sample names or other metadata.

In [11]:

```
chao1 = samples.alpha_diversity('chao1')
chao1['filename'] = [s.filename for s in samples]
```

```
#chao1['host_age_years'] = samples.metadata['host_age'][chao1.index] / 365
chao1
```

Out[11]:

classification_id	chao1	filename
99bce79f5f9e46c4	132.0	FS38906117.fastq.gz
e95a62fa8fa8421c	96.0	FS38906254.fastq.gz
1b5fb828156a45f1	197.0	FS38993450.fastq.gz
fb7a978eba07450b	204.0	FS38994104.fastq.gz
c1c3306bcaa242f4	156.0	FS68661606.fastq.gz
1d4a86f3b8de4419	142.0	FS68661649.fastq.gz
3b4230c2315e4b38	74.0	FS68661772.fastq.gz
b0b0eed901884e5c	92.0	FS68661818.fastq.gz
8eb32381cb5847cc	174.0	FS68662119.fastq.gz
63a194f3415e4040	242.0	FS68662229.fastq.gz
f2f980f0d74d48b1	41.0	FS68662318.fastq.gz
e3f9c8b57d624c2f	174.0	FS68662320.fastq.gz
141ae8562eac4d74	236.0	FS68662393.fastq.gz
4aaae28c3bb14949	245.0	FS68662593.fastq.gz
6a835d31633948ad	55.0	FS68662666.fastq.gz
732fa96a0a334f4f	51.0	FS68662793.fastq.gz
c338cd9070c84ac4	107.0	FS68669789.fastq.gz
65536da7db814d0e	95.0	FS68670071.fastq.gz
c04e8bb1ab814c56	152.0	FS68670134.fastq.gz
d67f23f904634686	125.0	FS68670142.fastq.gz
bf8040ed1e744f4f	45.0	FS38992039.fastq.gz
2fefd1c1040a4870	34.0	FS38994645.fastq.gz
19f9bf1124584bba	95.0	FS38994792.fastq.gz
64ce2d6507f24d36	44.0	FS38995936.fastq.gz
47916150098d4399	65.0	FS38996041.fastq.gz
5e474f22ddf44548	45.0	FS38996156.fastq.gz
6674e09cac224504	45.0	FS38996183.fastq.gz
d49be622f7c4440c	31.0	FS68624231.fastq.gz

4. Beta diversity statistics

Next, we'll explore a range of built-in beta diversity statistics. Most of these functions directly return a `skbio` distance matrix object.

In [12]:
```python
distance_matrix = samples.unifrac()
```

In [13]:
```python
type(distance_matrix)
```

Out[13]:
```
skbio.stats.distance._base.DistanceMatrix
```

In [14]:
```python
# Printing shows the distance matrix. Here, the labels are classification IDs.
distance_matrix.to_data_frame()
```

Out[14]:

	99bce79f5f9e46c4	e95a62fa8fa8421c	1b5fb828156a45f1	fb7a978eba07450
**99bce79f5f9e46c4**	0.000000	0.107376	0.209128	0.19670
**e95a62fa8fa8421c**	0.107376	0.000000	0.263864	0.25870
**1b5fb828156a45f1**	0.209128	0.263864	0.000000	0.14024
**fb7a978eba07450b**	0.196705	0.258700	0.140241	0.00000
**c1c3306bcaa242f4**	0.211810	0.284899	0.165134	0.26146
**1d4a86f3b8de4419**	0.122012	0.107143	0.272507	0.26018
**3b4230c2315e4b38**	0.209850	0.212196	0.243216	0.22819
**b0b0eed901884e5c**	0.135323	0.152847	0.241633	0.22368
**8eb32381cb5847cc**	0.139955	0.177544	0.148264	0.12192
**63a194f3415e4040**	0.226003	0.286873	0.070294	0.16409
**f2f980f0d74d48b1**	0.156029	0.139310	0.295248	0.27882
**e3f9c8b57d624c2f**	0.159654	0.162845	0.188561	0.12497
**141ae8562eac4d74**	0.188752	0.214952	0.152455	0.09096
**4aaae28c3bb14949**	0.218380	0.276252	0.072563	0.15538
**6a835d31633948ad**	0.159895	0.166753	0.279472	0.26753
**732fa96a0a334f4f**	0.152080	0.112379	0.289119	0.27814
**c338cd9070c84ac4**	0.148178	0.110241	0.297958	0.28315
**65536da7db814d0e**	0.197072	0.242356	0.200542	0.24921
**c04e8bb1ab814c56**	0.084898	0.124785	0.221284	0.21367
**d67f23f904634686**	0.252934	0.300750	0.194610	0.30065
**bf8040ed1e744f4f**	0.166997	0.202167	0.230468	0.25116
**2fefd1c1040a4870**	0.177146	0.134464	0.324406	0.31041
**19f9bf1124584bba**	0.217804	0.293208	0.174665	0.27520
**64ce2d6507f24d36**	0.161425	0.127963	0.304624	0.29129
**47916150098d4399**	0.169681	0.214229	0.186567	0.25391
**5e474f22ddf44548**	0.148944	0.119054	0.297940	0.28474
**6674e09cac224504**	0.141893	0.155644	0.262543	0.25644
**d49be622f7c4440c**	0.195797	0.158285	0.339590	0.31249

28 rows × 28 columns

5. Exploratory plots – beta diversity

Alternatively, we can use the built-in `plot_distance` function to visualize a distance matrix. The title of these plots can be set by passing the `title=` keyword argument.

In [15]:
```
samples.plot_distance(title='Bray Curtis Distance Matrix', metric='braycurtis',la
```

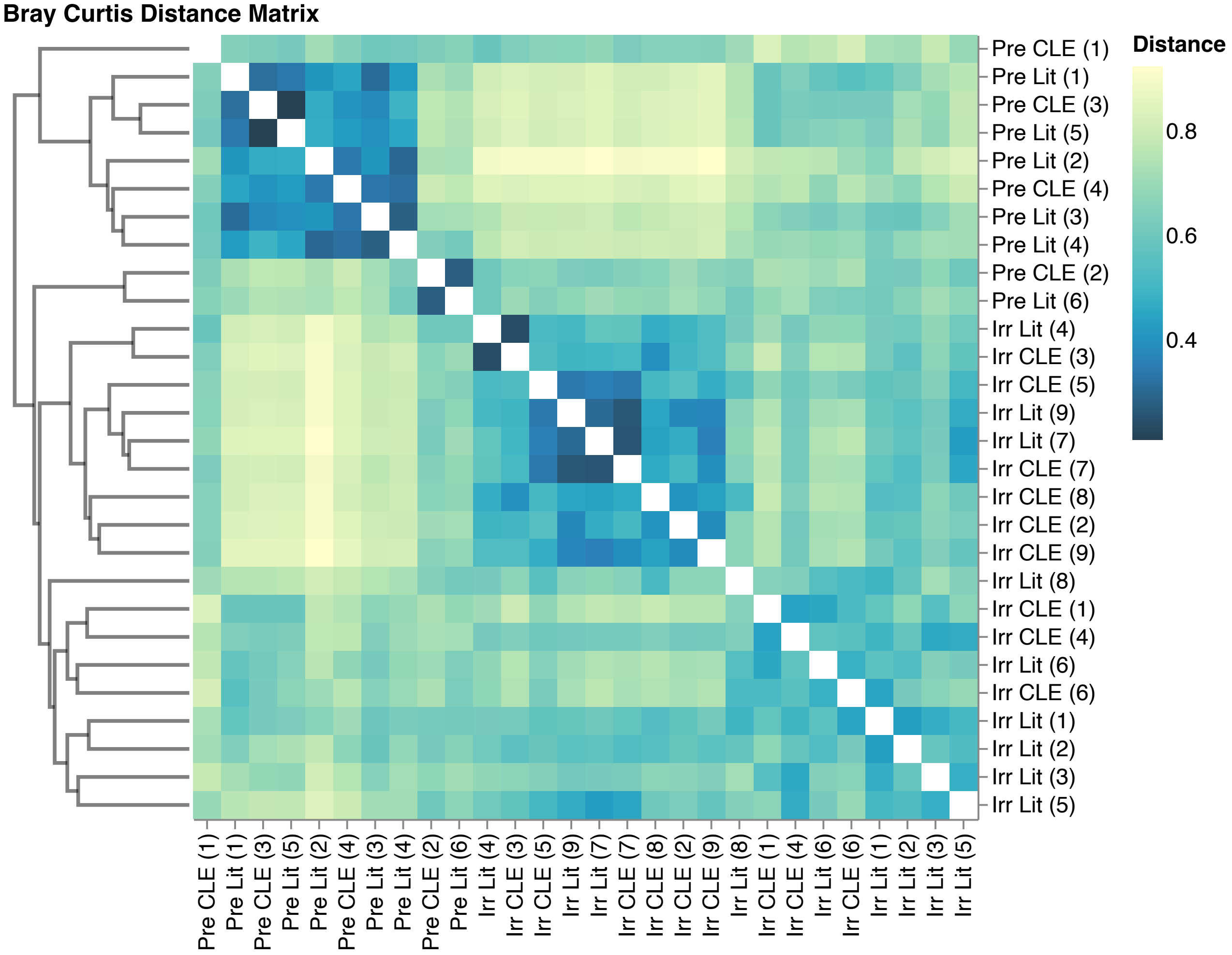

We can also plot other metrics, like weighted or unweighted Unifrac.

In [16]:
```
samples.plot_distance(title='Weighted Unifrac Distance Matrix', metric='weighted_
```

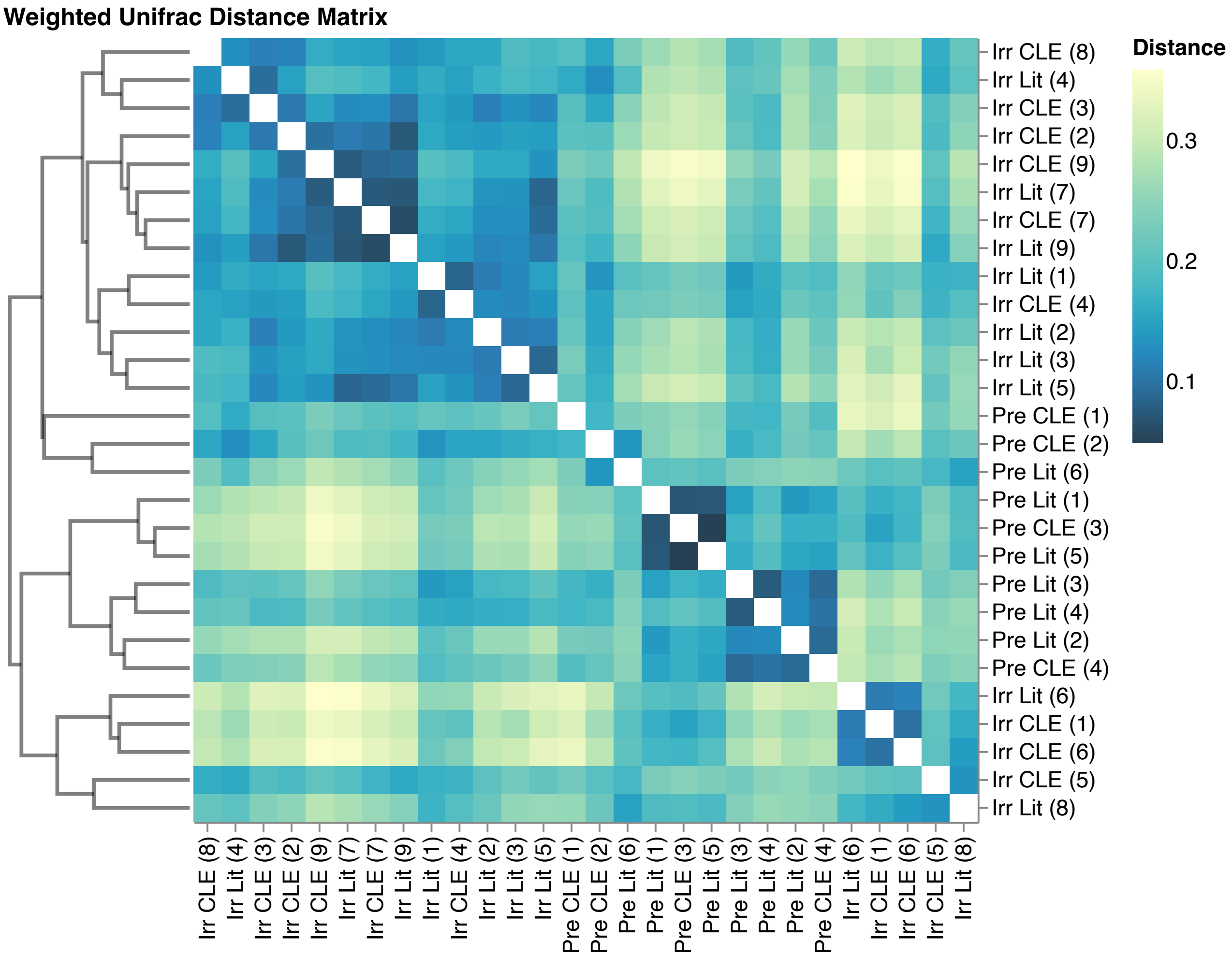

You can always call `help()` on any of the functions in our library for a detailed description of what they do, and a few examples!

In [17]:
```
help(samples.plot_distance)
```

```
Help on method plot_distance in module onecodex.viz._distance:

plot_distance(rank=<Rank.Auto: 'auto'>, metric=<BetaDiversityMetric.BrayCurtis: 
'braycurtis'>, title=None, xlabel=None, ylabel=None, tooltip=None, return_chart=F
alse, linkage=<Linkage.Average: 'average'>, label=None, width=None, height=None, 
match_taxonomy=True) method of onecodex.models.collection.SampleCollection instan
ce
    Plot beta diversity distance matrix as a heatmap and dendrogram.

    Parameters
    ----------
    rank : {'auto', 'kingdom', 'phylum', 'class', 'order', 'family', 'genus', 'sp
ecies'}, optional
        Analysis will be restricted to abundances of taxa at the specified level.
    metric : {'braycurtis', 'cityblock', 'manhattan', 'jaccard', 'unifrac', 'unwe
ighted_unifrac', 'aitchison'}, optional
        Function to use when calculating the distance between two samples.
        Note that 'cityblock' and 'manhattan' are equivalent metrics.
    linkage : {'average', 'single', 'complete', 'weighted', 'centroid', 'median'}
        The type of linkage to use when clustering axes.
    title : `string`, optional
        Text label at the top of the plot.
    xlabel : `string`, optional
        Text label along the horizontal axis.
    ylabel : `string`, optional
        Text label along the vertical axis.
    tooltip : `string` or `list`, optional
        A string or list containing strings representing metadata fields. When a 
point in the
        plot is hovered over, the value of the metadata associated with that samp
le will be
        displayed in a modal.
    label : `string` or `callable`, optional
        A metadata field (or function) used to label each analysis. If passing a 
function, a
        dict containing the metadata for each analysis is passed as the first and
only
        positional argument. The callable function must return a string.
    match_taxonomy : `bool`, default=True
        Whether or not to consider taxonomic names when looking for metadata fiel
ds mapped to
        plot attributes such as `tooltip`

    Examples
    --------
    Plot the weighted UniFrac distance between all our samples, using counts at t
he genus level.

    >>> samples.plot_distance(rank='genus', metric='unifrac')
```

6. Heatmaps

Our library includes support for plotting heatmaps. By default, heatmaps display the top 10 taxa, but this can be changed by passing `top_n=`. Alternatively, pass `threshold=cutoff` to display only taxa above a certain abundance threshold, or use a combination of both `threshold` and `top_n`. Pass, e.g., `rank='genus'` to restrict the analysis to a specific

taxonomic rank. As with `plot_distance`, you can customize the plot using `title=`. In addition, specify `xlabel=` or `ylabel=` to label the axes.

By default, all plots currently use the `readcount_w_children` value (reads at and below a taxonomic rank).

In [18]:
```
help(samples.plot_heatmap)
```

```
Help on method plot_heatmap in module onecodex.viz._heatmap:

plot_heatmap(rank=<Rank.Auto: 'auto'>, normalize='auto', top_n='auto', threshold
='auto', title=None, xlabel=None, ylabel=None, tooltip=None, return_chart=False,
linkage=<Linkage.Average: 'average'>, haxis=None, metric='euclidean', legend='aut
o', label=None, sort_x=None, sort_y=None, width=None, height=None, link=<Link.Oc
x: 'ocx'>, match_taxonomy=True) method of onecodex.models.collection.SampleCollec
tion instance
    Plot heatmap of taxa abundance/count data for several samples.

    Parameters
    ----------
    rank : {'auto', 'kingdom', 'phylum', 'class', 'order', 'family', 'genus', 'sp
ecies'}, optional
        Analysis will be restricted to abundances of taxa at the specified level.
    normalize : 'auto' or `bool`, optional
        Convert read counts to relative abundances such that each sample sums to
1.0. Setting
        'auto' will choose automatically based on the data.
    return_chart : `bool`, optional
        When True, return an `altair.Chart` object instead of displaying the resu
lting plot in
        the current notebook.
    haxis : `string`, optional
        The metadata field (or tuple containing multiple categorical fields) used
to group
        samples together. Each group of samples will be clustered independently.
    metric : {'euclidean', 'braycurtis', 'cityblock', 'manhattan', 'jaccard', 'un
ifrac', 'unweighted_unifrac', 'aitchison'}, optional
        Function to use when calculating the distance between two samples.
        Note that 'cityblock' and 'manhattan' are equivalent metrics.
    linkage : {'average', 'single', 'complete', 'weighted', 'centroid', 'median'}
        The type of linkage to use when clustering axes.
    top_n : `int`, optional
        Display the top N most abundant taxa in the entire cohort of samples.
    threshold : `float`
        Display only taxa that are more abundant that this threshold in one or mo
re samples.
    title : `string`, optional
        Text label at the top of the plot.
    xlabel : `string`, optional
        Text label along the horizontal axis.
    ylabel : `string`, optional
        Text label along the vertical axis.
    tooltip : `string` or `list`, optional
        A string or list containing strings representing metadata fields. When a
point in the
        plot is hovered over, the value of the metadata associated with that samp
le will be
        displayed in a modal.
    legend: `string`, optional
        Title for color scale. Defaults to the field used to generate the plot,
e.g.
        readcount_w_children or abundance.
    label : `string` or `callable`, optional
        A metadata field (or function) used to label each analysis. If passing a
function, a
        dict containing the metadata for each analysis is passed as the first and
only
        positional argument. The callable function must return a string.
```

```
    sort_x : `list` or `callable`, optional
        Either a list of sorted labels or a function that will be called with a l
ist of x-axis labels
        as the only argument, and must return the same list in a user-specified o
rder.
    sort_y : `list` or `callable`, optional
        Either a list of sorted labels or a function that will be called with a l
ist of y-axis labels
        as the only argument, and must return the same list in a user-specified o
rder.
    link : {'ocx', 'ncbi'}, optional
        If `link` is 'ocx', clicking a sample will open its classification result
s in the One
        Codex app. If `link` is 'ncbi', clicking a taxon will open the NCBI taxon
omy browser.
    match_taxonomy : `bool`, default=True
        Whether or not to consider taxonomic names when looking for metadata fiel
ds mapped to
        plot attributes including `tooltip`, `label`

    Examples
    --------
    Plot a heatmap of the relative abundances of the top 10 most abundant familie
s.

    >>> samples.plot_heatmap(rank='family', top_n=10)
```

In [19]:

```python
genera = samples.plot_heatmap(top_n=10,
    normalize= 'auto', rank='genus', title='Top 10 Genera', haxis='Status2',
    ylabel='Normalized Read Count', return_chart=True, legend="", label='Status2'
)
species = samples.plot_heatmap(top_n=10,
    normalize= 'auto', rank='species', title='Top 10 Species', haxis='Status2',
    ylabel='Normalized Read Count', return_chart=True, legend="", label='Status2'
)

genera | species
```

Out[19]:

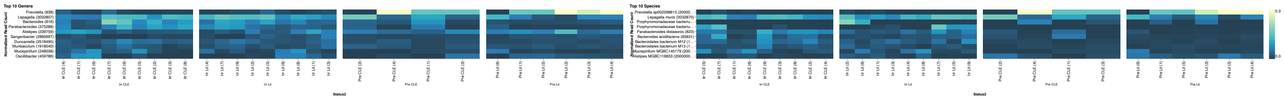

In [20]:

```python
sample_labels = samples.metadata['Status2'].tolist()


status_order = ["Pre Lit", "Pre CLE", "Irr Lit", "Irr CLE"]
```

In [21]:

```python
genera = samples.plot_heatmap(top_n=10,
    normalize= 'auto', rank='genus', title='Top 10 Genera',
    ylabel='Normalized Read Count', return_chart=True, legend="", label='Status2'
)
phyla = samples.plot_heatmap(top_n=10,
    normalize= 'auto', rank='phylum', title='Top 10 Phyla',
    ylabel='Normalized Read Count', return_chart=True, legend="", label='Status2'
)

genera | phyla
```

Out[21]:

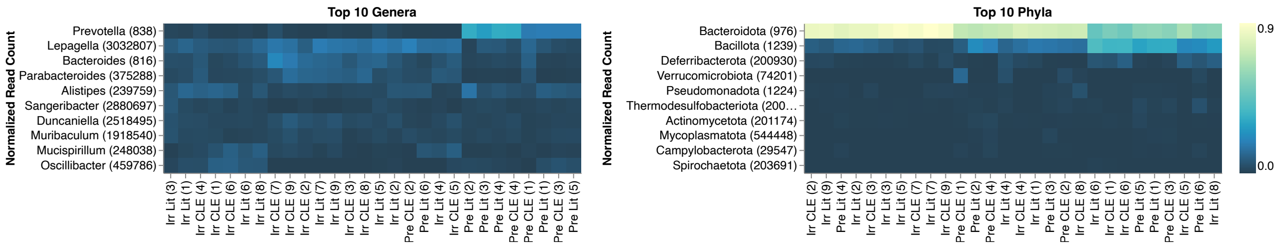

In [22]:

```
samples.metadata.Status2
```

Out[22]:

```
classification_id
99bce79f5f9e46c4    Irr Lit
e95a62fa8fa8421c    Irr Lit
1b5fb828156a45f1    Pre Lit
fb7a978eba07450b    Pre Lit
c1c3306bcaa242f4    Irr CLE
1d4a86f3b8de4419    Irr Lit
3b4230c2315e4b38    Pre CLE
b0b0eed901884e5c    Pre CLE
8eb32381cb5847cc    Pre Lit
63a194f3415e4040    Pre CLE
f2f980f0d74d48b1    Irr CLE
e3f9c8b57d624c2f    Pre Lit
141ae8562eac4d74    Pre CLE
4aaae28c3bb14949    Pre Lit
6a835d31633948ad    Irr Lit
732fa96a0a334f4f    Irr CLE
c338cd9070c84ac4    Irr Lit
65536da7db814d0e    Pre Lit
c04e8bb1ab814c56    Irr CLE
d67f23f904634686    Irr Lit
bf8040ed1e744f4f    Irr CLE
2fefd1c1040a4870    Irr Lit
19f9bf1124584bba    Irr CLE
64ce2d6507f24d36    Irr CLE
47916150098d4399    Irr Lit
5e474f22ddf44548    Irr Lit
6674e09cac224504    Irr CLE
d49be622f7c4440c    Irr CLE
Name: Status2, dtype: object
```

In [23]:

```
def sort_by_status2(sample_labels):
    # Define your desired Status2 order
    status_order = ["Pre Lit", "Pre CLE", "Irr Lit", "Irr CLE"]

    # Extract the status part (before the parentheses) and sort accordingly
    def get_status_order(label):
        # Extract the status part before the parentheses
        status = label.split(' (')[0]  # e.g., 'Irr CLE (5)' -> 'Irr CLE'
        return status_order.index(status) if status in status_order else len(stat

    return sorted(sample_labels, key=get_status_order)

genera = samples.plot_heatmap(top_n=10,
    normalize='auto', rank='genus', title='Top 10 Genera', sort_x=sort_by_status2
    ylabel='Normalized Read Count', return_chart=True, legend="", label='Status2'
)
phyla = samples.plot_heatmap(top_n=10,
    normalize='auto', rank='phylum', title='Top 10 Phyla', sort_x=sort_by_status2
    ylabel='Normalized Read Count', return_chart=True, legend="", label='Status2'
```

```python
)
genera | phyla
```

Out[23]:

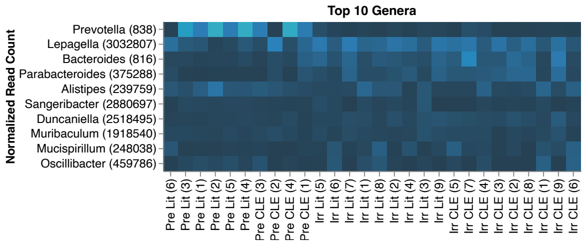

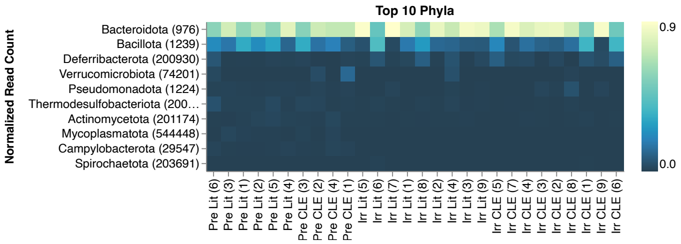

In [24]:

```python
def sort_by_status2(sample_labels):
    # Define your desired Status2 order
    status_order = ["Pre Lit", "Pre CLE", "Irr Lit", "Irr CLE"]

    # Extract the status part (before the parentheses) and sort accordingly
    def get_status_order(label):
        # Extract the status part before the parentheses
        status = label.split(' (')[0]  # e.g., 'Irr CLE (5)' -> 'Irr CLE'
        return status_order.index(status) if status in status_order else len(stat

    return sorted(sample_labels, key=get_status_order)

species = samples.plot_heatmap(top_n=10,
    normalize='auto', rank='species', title='Top 10 Species', sort_x=sort_by_stat
    ylabel='Normalized Read Count', return_chart=True, legend="", label='Status2'
)

species
```

Out[24]:

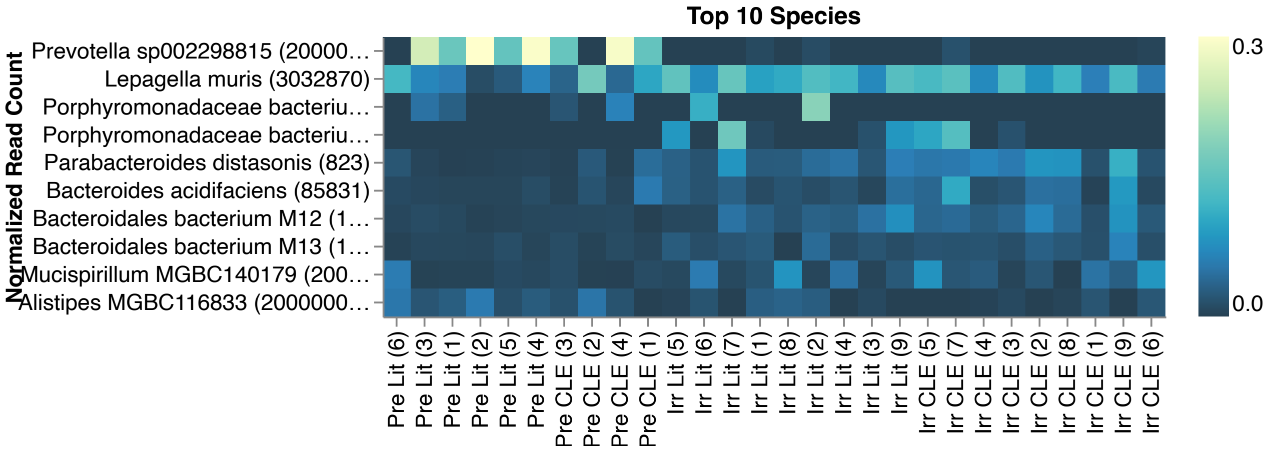

In [25]:

```python
print("Metadata columns:")
print(samples.metadata.columns.tolist())
print("\nFirst few rows of metadata:")
print(samples.metadata.head())
```

```
Metadata columns:
['date_collected', 'date_sequenced', 'description', 'external_sample_id', 'librar
y_type', 'location_lat', 'location_lon', 'location_string', 'name', 'platform',
'sample_type', 'starred', 'updated_at', 'sample_id', 'metadata_id', 'created_at',
'filename', 'project', 'Behavior testing', 'Behavioral testing: Acceleration time
test after 10 seconds', 'Brain weight', 'Cage', 'Cells Injected', 'Cohort', 'Day
s', 'Disease', 'Donor DOB', 'Donor age', 'Experiment', 'Experiment type', 'Experi
ment.1', 'Expt start date', 'Genotype status', 'IV or IP', 'Inject/non', 'Mouse I
D', 'Mouse ID.1', 'Mouse age', 'Mouse birth date', 'Mouse weight', 'Name.1', 'Ord
er 1', 'Order Number', 'Post Groups', 'Pre/Post Groups', 'Sample Origin', 'Scor
e', 'Score.1', 'Sex', 'Skin Score', 'Sort order', 'Speed-rpm', 'Spleen Weight',
'Spleen weight', 'Status', 'Status 1', 'Status2', 'T cell status', 'TRE-2 Tg', 'T
lr9-1 KO', 'Tlr9-1 WT', 'Treatment status', 'Weight', 'tRFP', 'tTA', 'Pre Group
s', 'Brain Weight', 'All Irradiated', 'Unnamed: 39', 'Unnamed: 40', 'Unnamed: 4
2', 'Unnamed: 43', 'Number Order 1', 'Rack']

First few rows of metadata:
                  date_collected date_sequenced  \
classification_id
99bce79f5f9e46c4            None           None
e95a62fa8fa8421c            None           None
1b5fb828156a45f1            None           None
fb7a978eba07450b            None           None
c1c3306bcaa242f4            None           None

                                                        description  \
classification_id
99bce79f5f9e46c4   CLE-10 mice before and 3 weeks after irradiati...
e95a62fa8fa8421c   CLE-10 mice before and 3 weeks after irradiati...
1b5fb828156a45f1   CLE-10 mice before and 3 weeks after irradiati...
fb7a978eba07450b   CLE-10 mice before and 3 weeks after irradiati...
c1c3306bcaa242f4   CLE-10 mice before and 3 weeks after irradiati...

                  external_sample_id library_type location_lat location_lon  \
classification_id
99bce79f5f9e46c4          FS38906117         None         None         None
e95a62fa8fa8421c          FS38906254         None         None         None
1b5fb828156a45f1          FS38993450         None         None         None
fb7a978eba07450b          FS38994104         None         None         None
c1c3306bcaa242f4          FS68661606         None         None         None

                  location_string            name         platform  ... tTA  \
classification_id                                                   ...
99bce79f5f9e46c4                   94L irradiated  Illumina NovaSeq  ...   -
e95a62fa8fa8421c                   94O irradiated  Illumina NovaSeq  ...   -
1b5fb828156a45f1                      94L healthy  Illumina NovaSeq  ...   -
fb7a978eba07450b                      94O healthy  Illumina NovaSeq  ...   -
c1c3306bcaa242f4                   93R irradiated  Illumina NovaSeq  ...   +

                      Pre Groups Brain Weight All Irradiated Unnamed: 39  \
classification_id
99bce79f5f9e46c4             NaN          NaN            NaN         NaN
e95a62fa8fa8421c             NaN          NaN            NaN         NaN
1b5fb828156a45f1   LittermatePre          NaN            NaN         NaN
fb7a978eba07450b   LittermatePre          NaN            NaN         NaN
c1c3306bcaa242f4             NaN        425.0            NaN         NaN

                  Unnamed: 40 Unnamed: 42 Unnamed: 43 Number Order 1  Rack
classification_id
99bce79f5f9e46c4          NaN         NaN         NaN            NaN   NaN
```

```
e95a62fa8fa8421c          NaN        NaN        NaN          NaN    NaN
1b5fb828156a45f1          NaN        NaN        NaN          NaN    NaN
fb7a978eba07450b          NaN        NaN        NaN          NaN    NaN
c1c3306bcaa242f4          NaN        NaN        NaN          NaN    NaN

[5 rows x 74 columns]
```

In [26]:
```python
def debug_sort_function(sample_labels):
    print("Labels the heatmap function expects:")
    print(sample_labels)
    print(f"Number of labels: {len(sample_labels)}")
    print(f"Type: {type(sample_labels)}")
    return sample_labels  # Return unchanged to avoid breaking the function

# Use this debug function to see what labels are expected
genera = samples.plot_heatmap(top_n=10,
    normalize='auto', rank='genus', title='Top 10 Genera', sort_x=debug_sort_func
    ylabel='Normalized Read Count', return_chart=True, legend="", label='Status2'
)
```

```
Labels the heatmap function expects:
['Irr Lit (5)', 'Pre Lit (6)', 'Pre Lit (3)', 'Irr Lit (6)', 'Irr CLE (5)', 'Pre
Lit (1)', 'Irr Lit (7)', 'Irr CLE (7)', 'Irr CLE (4)', 'Pre CLE (3)', 'Pre Lit
(2)', 'Pre Lit (5)', 'Irr Lit (1)', 'Irr CLE (3)', 'Irr CLE (2)', 'Irr Lit (8)',
'Pre Lit (4)', 'Irr CLE (8)', 'Irr Lit (2)', 'Irr CLE (1)', 'Pre CLE (2)', 'Pre C
LE (4)', 'Irr CLE (9)', 'Irr Lit (4)', 'Irr Lit (3)', 'Irr Lit (9)', 'Irr CLE
(6)', 'Pre CLE (1)']
Number of labels: 28
Type: <class 'list'>
```

7. Stacked bar plots

Bar plots are a common way to view taxonomic abundance data.

In [27]:
```python
genera2 = samples.plot_bargraph(
    rank='genus', legend='Genus', ylabel='Relative Abundance', top_n=25, group_by
)
species2 = samples.plot_bargraph(
    rank='species', legend='Species', ylabel='Relative Abundance', top_n=25, grou
)

genera2
species2

#order was edited in Vega Editor for publication
```

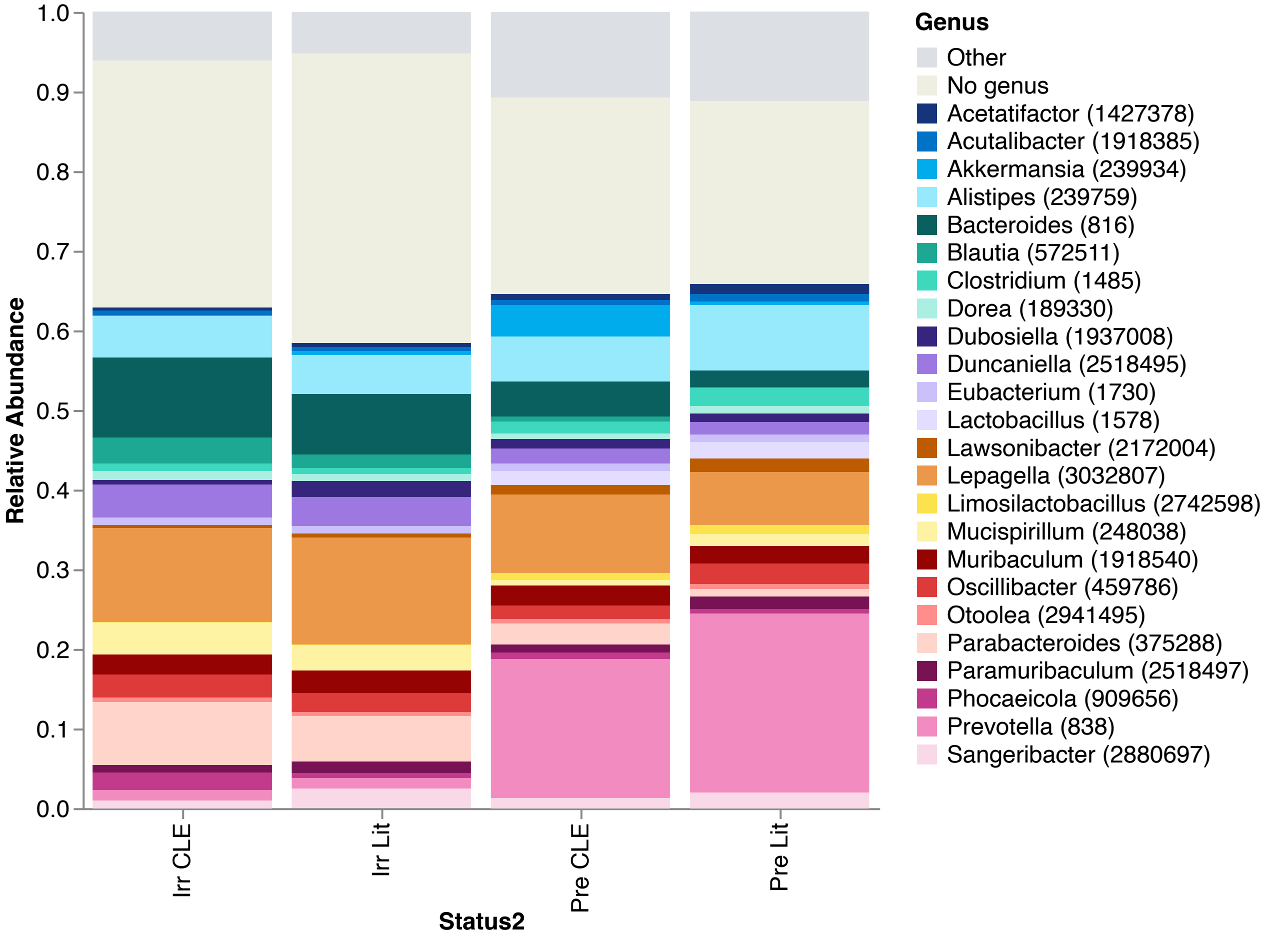

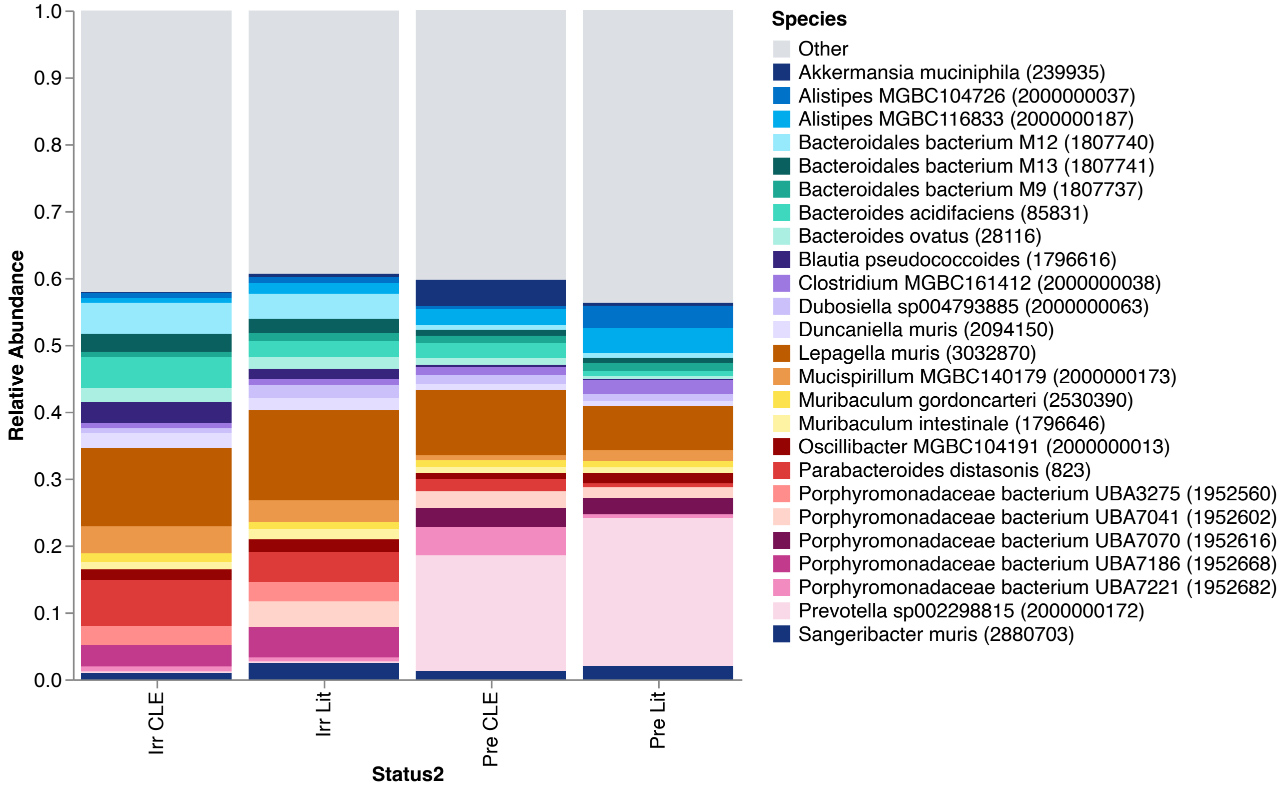

In [28]:

```
help(samples.plot_bargraph)
```

```
Help on method plot_bargraph in module onecodex.viz._bargraph:

plot_bargraph(rank=<Rank.Auto: 'auto'>, normalize='auto', top_n='auto', threshold
='auto', title=None, xlabel=None, ylabel=None, tooltip=None, return_chart=False,
haxis=None, legend='auto', label=None, sort_x=None, include_taxa_missing_rank=Non
e, include_other=True, width=None, height=None, group_by=None, link=<Link.Ocx: 'o
cx'>, match_taxonomy=True) method of onecodex.models.collection.SampleCollection
instance
    Plot a bargraph of relative abundance of taxa for multiple samples.

    Parameters
    ----------
    rank : {'auto', 'kingdom', 'phylum', 'class', 'order', 'family', 'genus', 'sp
ecies'}, optional
        Analysis will be restricted to abundances of taxa at the specified level.
    normalize : 'auto' or `bool`, optional
        Convert read counts to relative abundances such that each sample sums to
1.0. Setting
        'auto' will choose automatically based on the data.
    return_chart : `bool`, optional
        When True, return an `altair.Chart` object instead of displaying the resu
lting plot in
        the current notebook.
    top_n : `int`, optional
        Display the top N most abundant taxa in the entire cohort of samples.
    threshold : `float`
        Display only taxa that are more abundant that this threshold in one or mo
re samples.
    title : `string`, optional
        Text label at the top of the plot.
    xlabel : `string`, optional
        Text label along the horizontal axis.
    ylabel : `string`, optional
        Text label along the vertical axis.
    tooltip : `string` or `list`, optional
        A string or list containing strings representing metadata fields. When a
point in the
        plot is hovered over, the value of the metadata associated with that samp
le will be
        displayed in a modal.
    haxis : `string`, optional
        The metadata field (or tuple containing multiple categorical fields) used
to facet
        samples.
    legend: `string` or `altair.Legend`, optional
        If a string is provided, it will be used as the legend title. Defaults to
the metric
        used to generate the plot, e.g. readcount_w_children or abundance. Altern
atively, an
        `altair.Legend` instance may be provided for legend customization.
    label : `string` or `callable`, optional
        A metadata field (or function) used to label each analysis. If passing a
function, a
        dict containing the metadata for each analysis is passed as the first and
only
        positional argument. The callable function must return a string.
    sort_x : `list` or `callable`, optional
        Either a list of sorted labels or a function that will be called with a l
ist of x-axis labels
        as the only argument, and must return the same list in a user-specified o
```

```
rder.
    include_taxa_missing_rank : `bool`, optional
        Whether or not a row should be plotted for taxa that do not have a design
ated parent at `rank`.
    group_by : `string`, optional
        The metadata field used to group samples together. Readcounts or abundanc
es will be
        averaged within each group.
    link: {'ocx', 'ncbi'}, optional
        If `link` is 'ocx', clicking a sample will open its classification result
s in the One
        Codex app. If `link` is 'ncbi', clicking a taxon will open the NCBI taxon
omy browser.
    match_taxonomy : `bool`, default=True
        Whether or not to consider taxonomic names when looking for metadata fiel
ds mapped to
        plot attributes including `group_by`, `label`, `haxis`, & `tooltip`

    Examples
    --------
    Plot a bargraph of the top 10 most abundant genera

    >>> samples.plot_bargraph(rank='genus', top_n=10)
```

8. Ordination

Our library supports ordination via Principal Component Analysis (PCA) and multidimensional scaling (MDS, more commonly known as PCoA). Ordination is useful for exploring the similarity of large numbers of samples in a single plot. Points on these plots can be colored and/or sized by taxon abundance and metadata.

These plots are fully interactive--try zooming in on specific regions, or hovering over points to see the abundance of Firmicutes taxa in each sample!

In [29]:
```
help(samples.plot_pca)
```

```
Help on method plot_pca in module onecodex.viz._pca:

plot_pca(rank=<Rank.Auto: 'auto'>, normalize='auto', org_vectors=0, org_vectors_s
cale=None, title=None, xlabel=None, ylabel=None, color=None, size=None, tooltip=N
one, return_chart=False, label=None, mark_size=100, width=None, height=None, matc
h_taxonomy=True) method of onecodex.models.collection.SampleCollection instance
    Perform principal component analysis and plot first two axes.

    Parameters
    ----------
    rank : {'auto', 'kingdom', 'phylum', 'class', 'order', 'family', 'genus', 'sp
ecies'}, optional
        Analysis will be restricted to abundances of taxa at the specified level.
    normalize : 'auto' or `bool`, optional
        Convert read counts to relative abundances such that each sample sums to
1.0. Setting
        'auto' will choose automatically based on the data.
    org_vectors : `int`, optional
        Plot this many of the top-contributing eigenvectors from the PCA results.
    org_vectors_scale : `float`, optional
        Multiply the length of the lines representing the eigenvectors by this co
nstant.
    title : `string`, optional
        Text label at the top of the plot.
    xlabel : `string`, optional
        Text label along the horizontal axis.
    ylabel : `string`, optional
        Text label along the vertical axis.
    size : `string` or `tuple`, optional
        A string or a tuple containing strings representing metadata fields. The
size of points
        in the resulting plot will change based on the metadata associated with e
ach sample.
    color : `string` or `tuple`, optional
        A string or a tuple containing strings representing metadata fields. The
color of points
        in the resulting plot will change based on the metadata associated with e
ach sample.
    tooltip : `string` or `list`, optional
        A string or list containing strings representing metadata fields. When a
point in the
        plot is hovered over, the value of the metadata associated with that samp
le will be
        displayed in a modal.
    label : `string` or `callable`, optional
        A metadata field (or function) used to label each analysis. If passing a
function, a
        dict containing the metadata for each analysis is passed as the first and
only
        positional argument. The callable function must return a string.
    mark_size: `int`, optional
        The size of the points in the scatter plot.
    match_taxonomy : `bool`, default=True
        Whether or not to consider taxonomic names when looking for metadata fiel
ds mapped to
        plot attributes including `tooltip`, `color`, `label`, & `size`

    Examples
    --------
    Perform PCA on relative abundances at the species-level and color the resulti
```

```
ng points by
    'geo_loc_name', a metadata field representing the geographical origin of each
sample.

    >>> samples.plot_pca(rank='species', normalize=True, color='geo_loc_name')

    Change the size of each point in the plot based on the abundance of Bacteroid
es.

    >>> samples.plot_pca(size='Bacteroides')

    Display the abundances of Bacteroides, Prevotella, and Bifidobacterium in eac
h sample when
    hovering over points in the plot.

    >>> samples.plot_pca(tooltip=['Bacteroides', 'Prevotella', 'Bifidobacteriu
m'])
```

In [30]:

```
#samples.plot_pca(rank='species', color='Status2')
# size='Bacteroides', tooltip='Bacillota'

#samples.plot_pca(rank='species', normalize=True, color='Status2')

samples.plot_pca(color="Status2", size="Bacteroides")
```

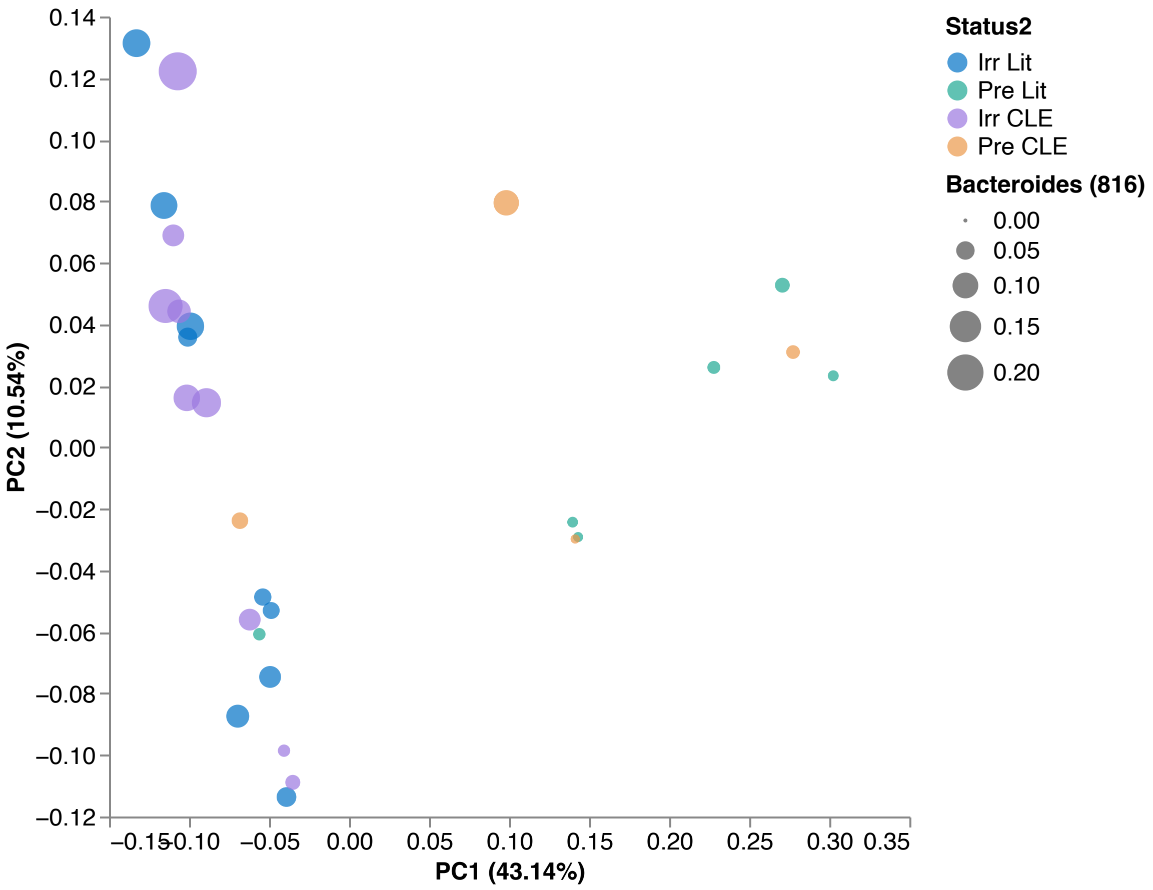

In [31]:

```
#samples.plot_pca(rank='species', color='Status2')
# size='Bacteroides', tooltip='Bacillota'

#samples.plot_pca(rank='species', normalize=True, color='Status2')
```

```
samples.plot_pca(color="Status2", size="Bacteroides")
samples.plot_pca(color="Inject/non", size="Bacteroides")
```

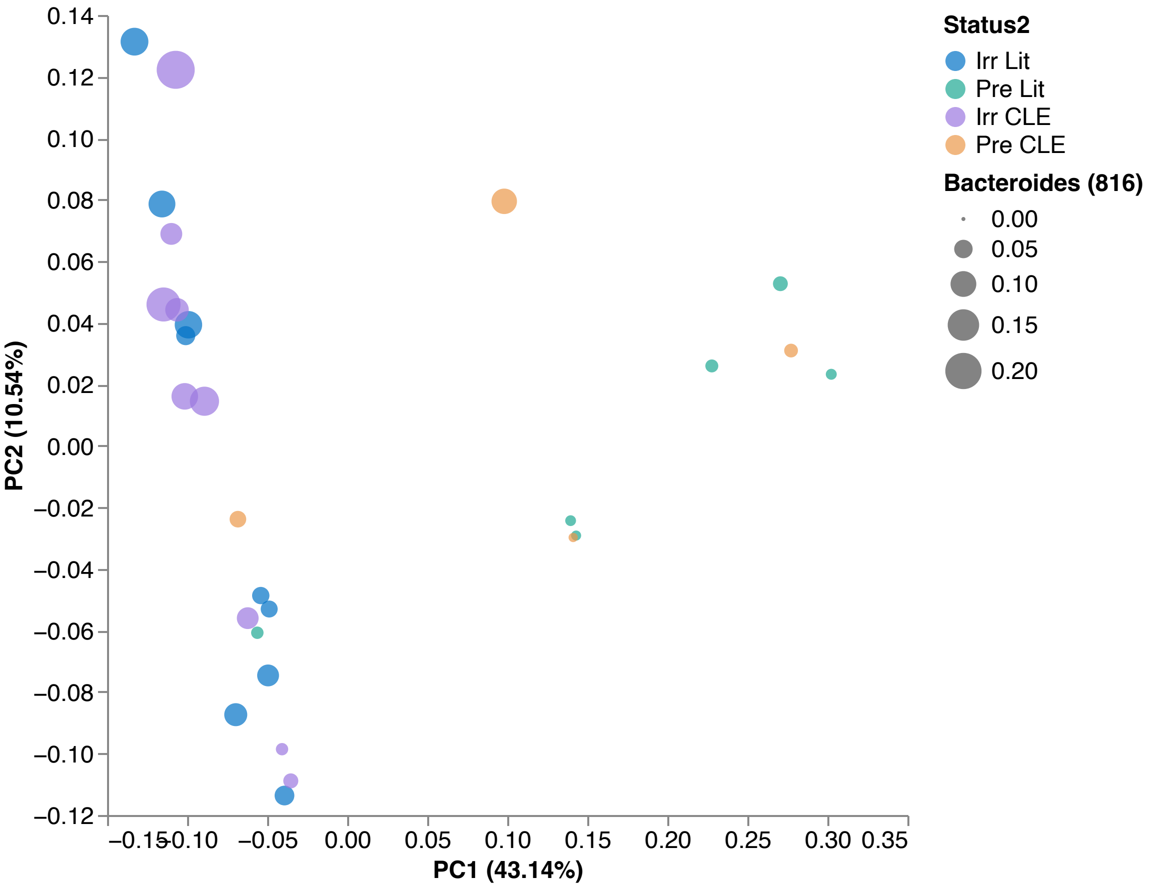

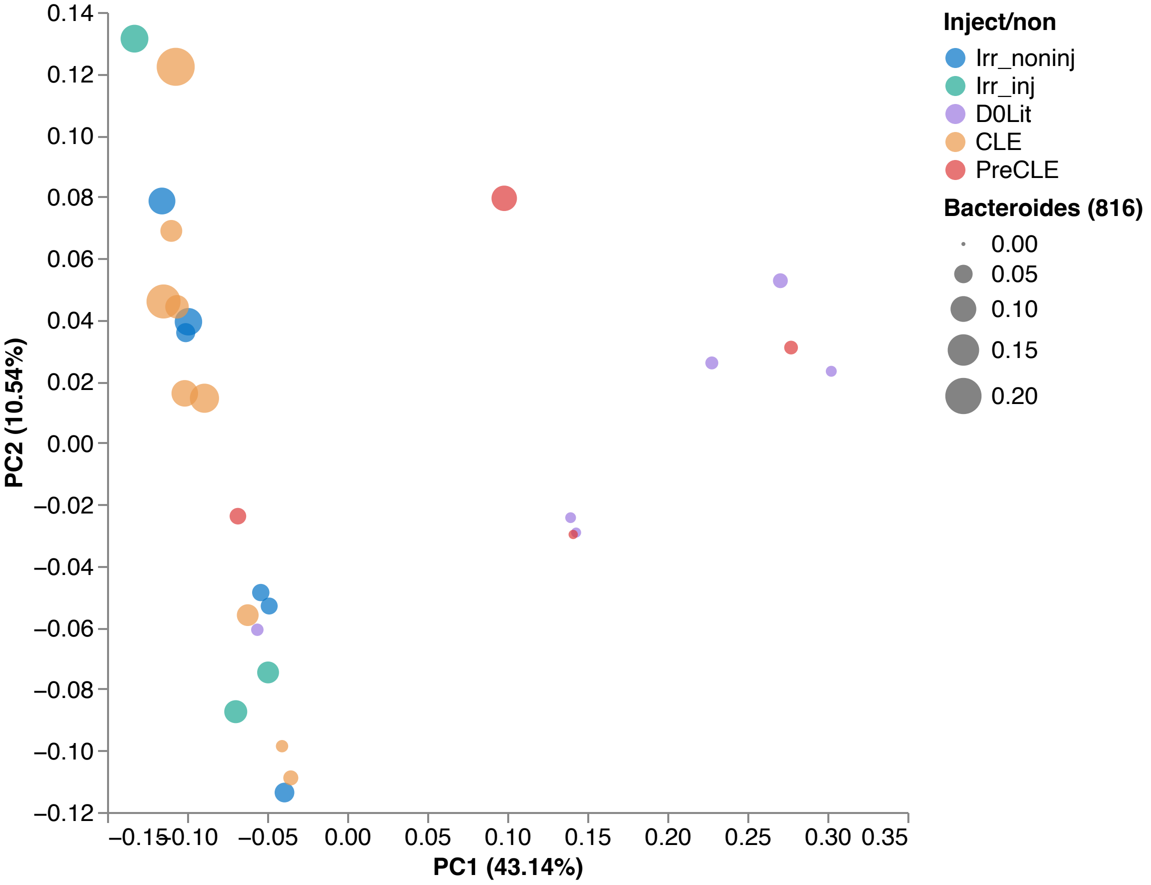

In [32]:
```python
# The 'smacof' algorithm performs MDS using iterative optimization
samples.plot_mds(rank= 'species',
    metric='weighted_unifrac', method='smacof', color='Status2',
    size='Bacteroides', tooltip='Bacillota'
)
```

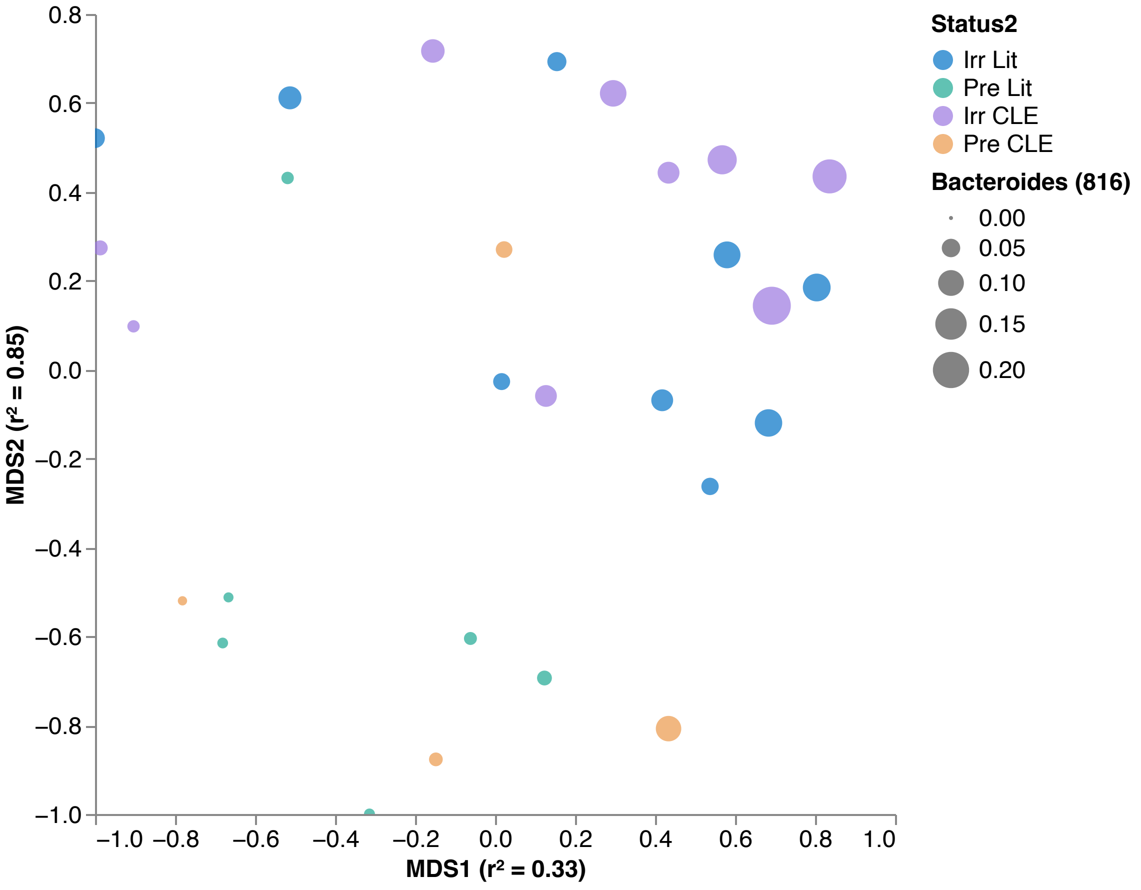

That's it! Please get in touch at hello@onecodex.com if you have questions or would like to sequence samples for a new project
