# Supplementary material for: Gut dysbiosis in a murine model of cutaneous lupus erythematosus correlates with antigen-specific T cells and antigen-presenting cells in skin
Source: Sci Rep. 2026 Jan 12;16:4511. doi: 10.1038/s41598-025-34741-6 (PMC12864916; doi:10.1038/s41598-025-34741-6)
Supplement: Supplementary file 4 — Supplementary Material 4 [file 41598_2025_34741_MOESM4_ESM.pdf]

Introduction

This notebook contains all of the code from the corresponding post on the One Codex Blog.

Useful resources: notes on getting started with our One Codex library; the full documentation on our API (more technical); a cheat sheet on getting started with Pandas, a Python library for data manipulation; and reading a few of our blog posts (where we plan to have nice demos with these notebooks).

Fetching data

To get started, we create an instance of our API and grab the Gut Microbiome project samples.

In [1]:
```
from onecodex import Api

ocx = Api()
```

In [2]:
```
ocx.Projects.where(name='Gut microbiome')

project = ocx.Projects.get('120f7b4173934b33')


#samples.metadata
```

In [3]:
```
ocx.Samples.where(project=project)
```

Out[3]:
```
[<Samples b4f2a64b8ea540bb: "FS38906117.fastq.gz">, <Samples fa97ff9f2613408d: "F
S38906254.fastq.gz">, <Samples 0d717ee658504d64: "FS38993450.fastq.gz">, <Samples
0f63f4eb6b824530: "FS38994104.fastq.gz">, <Samples 874241b8d6774308: "FS68661606.
fastq.gz">, <Samples 88d3301cb5c94c10: "FS68661649.fastq.gz">, <Samples e1d4e0f42
9ea48da: "FS68661772.fastq.gz">, <Samples 4a3ace12e4eb4933: "FS68661818.fastq.g
z">, <Samples f9e560be678b4672: "FS68662119.fastq.gz">, <Samples e09fdf89876e416
f: "FS68662229.fastq.gz">, <Samples 007de7018e564b0e: "FS68662318.fastq.gz">, <Sa
mples b966ab22fc02460d: "FS68662320.fastq.gz">, <Samples 3c7b5fe6cee24add: "FS686
62393.fastq.gz">, <Samples 01c115ace07c41be: "FS68662593.fastq.gz">, <Samples 7c7
c4590b13b4032: "FS68662666.fastq.gz">, <Samples f99e271bd1c74179: "FS68662793.fas
tq.gz">, <Samples eef6a5c0c6654e2c: "FS68669789.fastq.gz">, <Samples 9043dcaa0b36
4510: "FS68670071.fastq.gz">, <Samples 790e8999dd2c4ef2: "FS68670134.fastq.gz">,
<Samples 552f1847d539483f: "FS68670142.fastq.gz">, <Samples 1343388a7512471c: "FS
38992039.fastq.gz">, <Samples 80123f524edd48ea: "FS38994645.fastq.gz">, <Samples
c2b7514503f8405a: "FS38994792.fastq.gz">, <Samples 7235873cf8424846: "FS38995936.
fastq.gz">, <Samples 7f2d0c253ca644a8: "FS38996041.fastq.gz">, <Samples e5fff06dd
f9948f0: "FS38996156.fastq.gz">, <Samples a342d2287fe4438f: "FS38996183.fastq.g
z">, <Samples 76376a4abda74fe1: "FS68624231.fastq.gz">]
```

In [5]:
```
samples = ocx.Samples.where(project=project, limit=50)
```

Alpha diversity and Beta Diversity

Chao1, Simpson's Index, and Shannon Entropy side-by-side, grouped by disease status.

In [6]:

```
help(samples.plot_metadata)
```

```
Help on method plot_metadata in module onecodex.viz._metadata:

plot_metadata(rank=<Rank.Auto: 'auto'>, haxis='Label', vaxis=<AlphaDiversityMetri
c.Shannon: 'shannon'>, title=None, xlabel=None, ylabel=None, return_chart=False,
plot_type=<PlotType.Auto: 'auto'>, label=None, sort_x=None, width: Union[int, Lit
eral['container'], NoneType] = 200, height: Union[int, Literal['container'], None
Type] = 400, facet_by=None, coerce_haxis_dates=True, secondary_haxis=None, match_
taxonomy=True) method of onecodex.models.collection.SampleCollection instance
    Plot an arbitrary metadata field versus an arbitrary quantity as a boxplot or
scatter plot.

    Parameters
    ----------
    rank : {'auto', 'kingdom', 'phylum', 'class', 'order', 'family', 'genus', 'sp
ecies'}, optional
        Analysis will be restricted to abundances of taxa at the specified level.

    haxis : `string`, optional
        The metadata field (or tuple containing multiple categorical fields) to b
e plotted on
        the horizontal axis.

    vaxis : `string`, optional
        Data to be plotted on the vertical axis. Can be any one of the following:

        - A metadata field: the name of a metadata field containing numerical dat
a
        - {'simpson', 'observed_taxa', 'shannon'}: an alpha diversity statistic t
o calculate for
          each sample. Note that Shannon diversity is calculated using log base `
`e`` (natural
          log).
        - A taxon name: the name of a taxon in the analysis
        - A taxon ID: the ID of a taxon in the analysis

    title : `string`, optional
        Text label at the top of the plot.

    xlabel : `string`, optional
        Text label along the horizontal axis.

    ylabel : `string`, optional
        Text label along the vertical axis.

    plot_type : {'auto', 'boxplot', 'scatter'}
        By default, will determine plot type automatically based on the data. Oth
erwise, specify
        one of 'boxplot' or 'scatter' to set the type of plot manually.

    label : `string` or `callable`, optional
        A metadata field (or function) used to label each analysis. If passing a
function, a
        dict containing the metadata for each analysis is passed as the first and
only
        positional argument. The callable function must return a string.

    sort_x : `list` or `callable`, optional
        Either a list of sorted labels or a function that will be called with a l
ist of x-axis labels
        as the only argument, and must return the same list in a user-specified o
```

```
rder.

    width : `int` or `str`, optional
        Sets `altair.Chart.width`. If `"container"`, chart width will respond to
the width of
        the HTML container it is rendered in.

    height : `int` or `str`, optional
        Sets `altair.Chart.height`. If `"container"`, chart height will respond t
o the height of
        the HTML container it is rendered in.

    facet_by : `string`, optional
        The metadata field used to facet samples by (i.e. to create a separate su
bplot for each
        group of samples).

    coerce_haxis_dates : `bool`, optional
        If ``True``, ``haxis`` field name(s) containing the word "date" (after sp
litting on
        underscores) will be coerced to datetime dtype. For example, the field "d
ate_collected"
        will be coerced if ``coerce_haxis_dates`` is ``True``.

    secondary_haxis : str or tuple of str, optional
        The secondary metadata field (or tuple containing multiple categorical fi
elds) to be
        plotted on the horizontal axis.

    match_taxonomy : `bool`, default=True
        Whether or not to consider taxonomic names when looking for metadata fiel
ds mapped to
        plot attributes including `vaxis`, `haxis`, `secondary_axis`, `facet_by`,
& `label`

    Examples
    --------
    Generate a boxplot of the abundance of Bacteroides (genus) of samples grouped
by whether the
    individuals are allergic to dogs, cats, both, or neither.

    >>> plot_metadata(haxis=('allergy_dogs', 'allergy_cats'), vaxis='Bacteroide
s')
```

In [8]:

```
chao1 = samples.plot_metadata(vaxis="chao1", haxis="Status2", return_chart=True)
simpson = samples.plot_metadata(vaxis="simpson", haxis="Status2", return_chart=Tr
shannon = samples.plot_metadata(vaxis="shannon", haxis="Status2", return_chart=Tr

chao1 | simpson | shannon

#Edited order in Vega editor for manuscript
```

Out[8]:

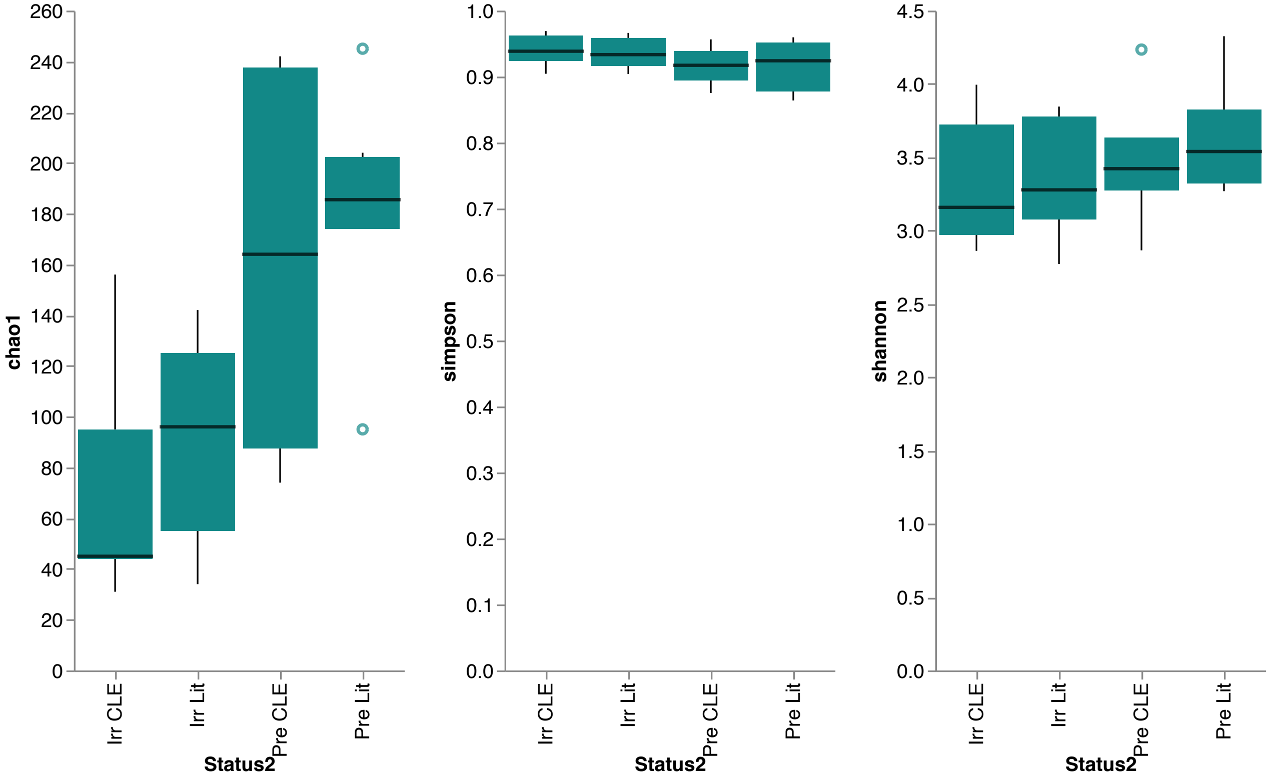

Distance Matrices and Metadata plotting

The `plot_metadata` function can search through all taxa in your samples and pull out read counts or relative abundances.

In [9]:
```
help(samples.plot_metadata)
```

```
Help on method plot_metadata in module onecodex.viz._metadata:

plot_metadata(rank=<Rank.Auto: 'auto'>, haxis='Label', vaxis=<AlphaDiversityMetri
c.Shannon: 'shannon'>, title=None, xlabel=None, ylabel=None, return_chart=False,
plot_type=<PlotType.Auto: 'auto'>, label=None, sort_x=None, width: Union[int, Lit
eral['container'], NoneType] = 200, height: Union[int, Literal['container'], None
Type] = 400, facet_by=None, coerce_haxis_dates=True, secondary_haxis=None, match_
taxonomy=True) method of onecodex.models.collection.SampleCollection instance
    Plot an arbitrary metadata field versus an arbitrary quantity as a boxplot or
scatter plot.

    Parameters
    ----------
    rank : {'auto', 'kingdom', 'phylum', 'class', 'order', 'family', 'genus', 'sp
ecies'}, optional
        Analysis will be restricted to abundances of taxa at the specified level.

    haxis : `string`, optional
        The metadata field (or tuple containing multiple categorical fields) to b
e plotted on
        the horizontal axis.

    vaxis : `string`, optional
        Data to be plotted on the vertical axis. Can be any one of the following:

        - A metadata field: the name of a metadata field containing numerical dat
a
        - {'simpson', 'observed_taxa', 'shannon'}: an alpha diversity statistic t
o calculate for
          each sample. Note that Shannon diversity is calculated using log base `
`e`` (natural
          log).
        - A taxon name: the name of a taxon in the analysis
        - A taxon ID: the ID of a taxon in the analysis

    title : `string`, optional
        Text label at the top of the plot.

    xlabel : `string`, optional
        Text label along the horizontal axis.

    ylabel : `string`, optional
        Text label along the vertical axis.

    plot_type : {'auto', 'boxplot', 'scatter'}
        By default, will determine plot type automatically based on the data. Oth
erwise, specify
        one of 'boxplot' or 'scatter' to set the type of plot manually.

    label : `string` or `callable`, optional
        A metadata field (or function) used to label each analysis. If passing a
function, a
        dict containing the metadata for each analysis is passed as the first and
only
        positional argument. The callable function must return a string.

    sort_x : `list` or `callable`, optional
        Either a list of sorted labels or a function that will be called with a l
ist of x-axis labels
        as the only argument, and must return the same list in a user-specified o
```

```
rder.

    width : `int` or `str`, optional
        Sets `altair.Chart.width`. If `"container"`, chart width will respond to
the width of
        the HTML container it is rendered in.

    height : `int` or `str`, optional
        Sets `altair.Chart.height`. If `"container"`, chart height will respond t
o the height of
        the HTML container it is rendered in.

    facet_by : `string`, optional
        The metadata field used to facet samples by (i.e. to create a separate su
bplot for each
        group of samples).

    coerce_haxis_dates : `bool`, optional
        If ``True``, ``haxis`` field name(s) containing the word "date" (after sp
litting on
        underscores) will be coerced to datetime dtype. For example, the field "d
ate_collected"
        will be coerced if ``coerce_haxis_dates`` is ``True``.

    secondary_haxis : str or tuple of str, optional
        The secondary metadata field (or tuple containing multiple categorical fi
elds) to be
        plotted on the horizontal axis.

    match_taxonomy : `bool`, default=True
        Whether or not to consider taxonomic names when looking for metadata fiel
ds mapped to
        plot attributes including `vaxis`, `haxis`, `secondary_axis`, `facet_by`,
& `label`

    Examples
    --------
    Generate a boxplot of the abundance of Bacteroides (genus) of samples grouped
by whether the
    individuals are allergic to dogs, cats, both, or neither.

    >>> plot_metadata(haxis=('allergy_dogs', 'allergy_cats'), vaxis='Bacteroide
s')
```

In [10]:
```
distance_matrix = samples.unifrac()
```

In [11]:
```
type(distance_matrix)
```

Out[11]:
```
skbio.stats.distance._base.DistanceMatrix
```

In [12]:
```
distance_matrix.to_data_frame()
```

Out[12]:

	99bce79f5f9e46c4	e95a62fa8fa8421c	1b5fb828156a45f1	fb7a978eba07450
**99bce79f5f9e46c4**	0.000000	0.107376	0.209128	0.19670
**e95a62fa8fa8421c**	0.107376	0.000000	0.263864	0.25870
**1b5fb828156a45f1**	0.209128	0.263864	0.000000	0.14024
**fb7a978eba07450b**	0.196705	0.258700	0.140241	0.00000
**c1c3306bcaa242f4**	0.211810	0.284899	0.165134	0.26146
**1d4a86f3b8de4419**	0.122012	0.107143	0.272507	0.26018
**3b4230c2315e4b38**	0.209850	0.212196	0.243216	0.22819
**b0b0eed901884e5c**	0.135323	0.152847	0.241633	0.22368
**8eb32381cb5847cc**	0.139955	0.177544	0.148264	0.12192
**63a194f3415e4040**	0.226003	0.286873	0.070294	0.16409
**f2f980f0d74d48b1**	0.156029	0.139310	0.295248	0.27882
**e3f9c8b57d624c2f**	0.159654	0.162845	0.188561	0.12497
**141ae8562eac4d74**	0.188752	0.214952	0.152455	0.09096
**4aaae28c3bb14949**	0.218380	0.276252	0.072563	0.15538
**6a835d31633948ad**	0.159895	0.166753	0.279472	0.26753
**732fa96a0a334f4f**	0.152080	0.112379	0.289119	0.27814
**c338cd9070c84ac4**	0.148178	0.110241	0.297958	0.28315
**65536da7db814d0e**	0.197072	0.242356	0.200542	0.24921
**c04e8bb1ab814c56**	0.084898	0.124785	0.221284	0.21367
**d67f23f904634686**	0.252934	0.300750	0.194610	0.30065
**bf8040ed1e744f4f**	0.166997	0.202167	0.230468	0.25116
**2fefd1c1040a4870**	0.177146	0.134464	0.324406	0.31041
**19f9bf1124584bba**	0.217804	0.293208	0.174665	0.27520
**64ce2d6507f24d36**	0.161425	0.127963	0.304624	0.29129
**47916150098d4399**	0.169681	0.214229	0.186567	0.25391
**5e474f22ddf44548**	0.148944	0.119054	0.297940	0.28474
**6674e09cac224504**	0.141893	0.155644	0.262543	0.25644
**d49be622f7c4440c**	0.195797	0.158285	0.339590	0.31249

28 rows × 28 columns

In [11]:

```
help(len)
```

```
Help on built-in function len in module builtins:

len(obj, /)
    Return the number of items in a container.
```

```
In [12]: len(samples)
```

```
Out[12]: 28
```

```
In [13]: help(samples.plot_distance)
```

```
Help on method plot_distance in module onecodex.viz._distance:

plot_distance(rank=<Rank.Auto: 'auto'>, metric=<BetaDiversityMetric.BrayCurtis:
'braycurtis'>, title=None, xlabel=None, ylabel=None, tooltip=None, return_chart=F
alse, linkage=<Linkage.Average: 'average'>, label=None, width=None, height=None,
match_taxonomy=True) method of onecodex.models.collection.SampleCollection instan
ce
    Plot beta diversity distance matrix as a heatmap and dendrogram.

    Parameters
    ----------
    rank : {'auto', 'kingdom', 'phylum', 'class', 'order', 'family', 'genus', 'sp
ecies'}, optional
        Analysis will be restricted to abundances of taxa at the specified level.
    metric : {'braycurtis', 'cityblock', 'manhattan', 'jaccard', 'unifrac', 'unwe
ighted_unifrac', 'aitchison'}, optional
        Function to use when calculating the distance between two samples.
        Note that 'cityblock' and 'manhattan' are equivalent metrics.
    linkage : {'average', 'single', 'complete', 'weighted', 'centroid', 'median'}
        The type of linkage to use when clustering axes.
    title : `string`, optional
        Text label at the top of the plot.
    xlabel : `string`, optional
        Text label along the horizontal axis.
    ylabel : `string`, optional
        Text label along the vertical axis.
    tooltip : `string` or `list`, optional
        A string or list containing strings representing metadata fields. When a
point in the
        plot is hovered over, the value of the metadata associated with that samp
le will be
        displayed in a modal.
    label : `string` or `callable`, optional
        A metadata field (or function) used to label each analysis. If passing a
function, a
        dict containing the metadata for each analysis is passed as the first and
only
        positional argument. The callable function must return a string.
    match_taxonomy : `bool`, default=True
        Whether or not to consider taxonomic names when looking for metadata fiel
ds mapped to
        plot attributes such as `tooltip`

    Examples
    --------
    Plot the weighted UniFrac distance between all our samples, using counts at t
he genus level.

    >>> samples.plot_distance(rank='genus', metric='unifrac')
```

```
In [19]: samples.plot_distance(title='Weighted Unifrac Distance Matrix', metric='weighted_
```

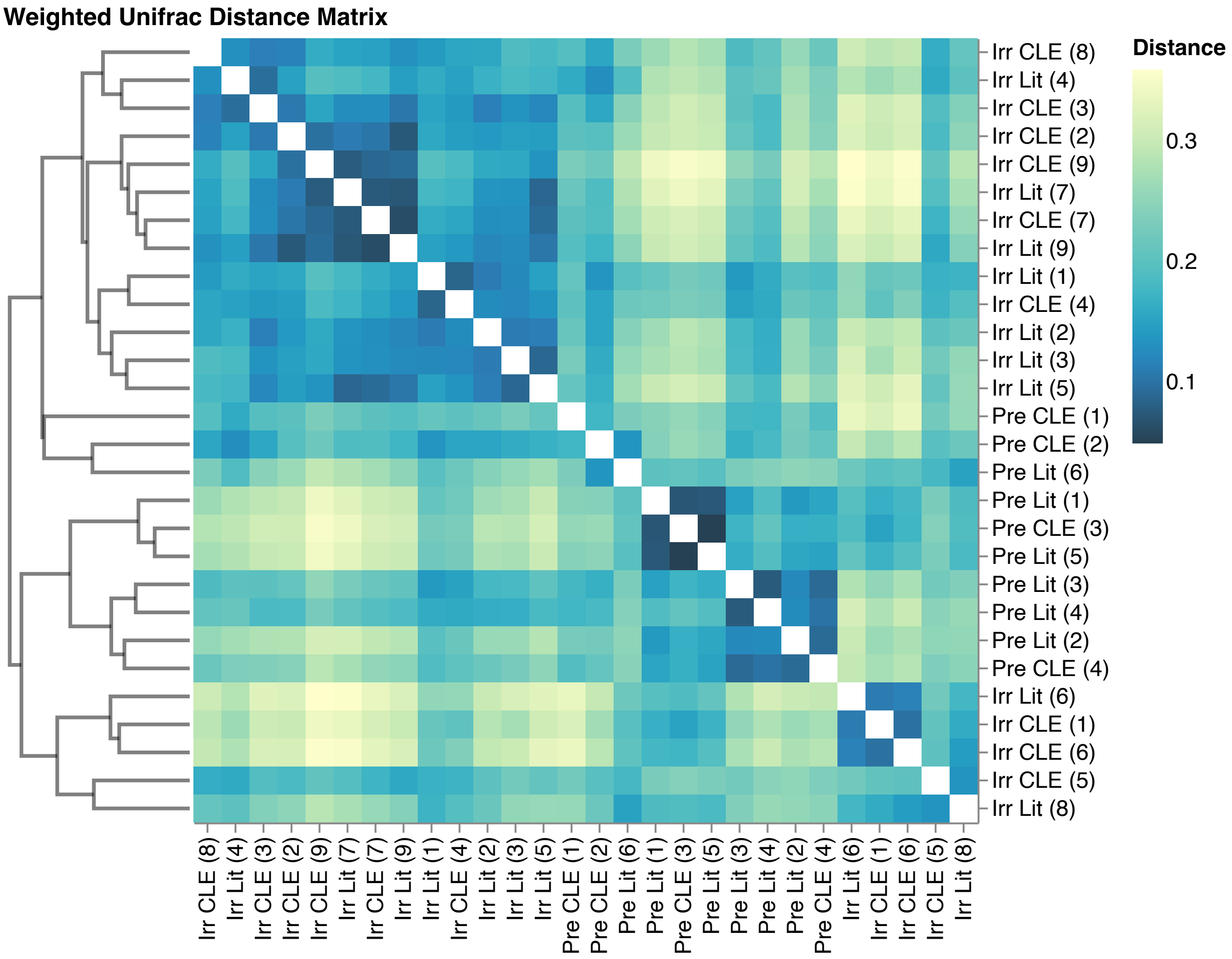

Heatmaps

In [13]:
```
#help(df_rel)
    #ocx.plot_heatmap)

help(samples.to_df)
```

```
Help on method to_df in module onecodex.analyses:

to_df(analysis_type=<AnalysisType.Classification: 'classification'>, **kwargs) me
thod of onecodex.models.collection.SampleCollection instance
    Transform Analyses of samples in a `SampleCollection` into tabular format.

    Parameters
    ----------
    analysis_type : {'classification', 'functional'}, optional
        The `analysis_type` to aggregate, corresponding to AnalysisJob.analysis_t
ype
    kwargs : dict, optional
         Keyword arguments specific to the `analysis_type`; see each individual f
unction definition

    .. seealso:: to_classification_df
    .. seealso:: to_functional_df
```

In [14]:
```
samples
```

Out[14]:

```
[<Samples b4f2a64b8ea540bb: "FS38906117.fastq.gz">, <Samples fa97ff9f2613408d: "F
S38906254.fastq.gz">, <Samples 0d717ee658504d64: "FS38993450.fastq.gz">, <Samples
0f63f4eb6b824530: "FS38994104.fastq.gz">, <Samples 874241b8d6774308: "FS68661606.
fastq.gz">, <Samples 88d3301cb5c94c10: "FS68661649.fastq.gz">, <Samples e1d4e0f42
9ea48da: "FS68661772.fastq.gz">, <Samples 4a3ace12e4eb4933: "FS68661818.fastq.g
z">, <Samples f9e560be678b4672: "FS68662119.fastq.gz">, <Samples e09fdf89876e416
f: "FS68662229.fastq.gz">, <Samples 007de7018e564b0e: "FS68662318.fastq.gz">, <Sa
mples b966ab22fc02460d: "FS68662320.fastq.gz">, <Samples 3c7b5fe6cee24add: "FS686
62393.fastq.gz">, <Samples 01c115ace07c41be: "FS68662593.fastq.gz">, <Samples 7c7
c4590b13b4032: "FS68662666.fastq.gz">, <Samples f99e271bd1c74179: "FS68662793.fas
tq.gz">, <Samples eef6a5c0c6654e2c: "FS68669789.fastq.gz">, <Samples 9043dcaa0b36
4510: "FS68670071.fastq.gz">, <Samples 790e8999dd2c4ef2: "FS68670134.fastq.gz">,
<Samples 552f1847d539483f: "FS68670142.fastq.gz">, <Samples 1343388a7512471c: "FS
38992039.fastq.gz">, <Samples 80123f524edd48ea: "FS38994645.fastq.gz">, <Samples
c2b7514503f8405a: "FS38994792.fastq.gz">, <Samples 7235873cf8424846: "FS38995936.
fastq.gz">, <Samples 7f2d0c253ca644a8: "FS38996041.fastq.gz">, <Samples e5fff06dd
f9948f0: "FS38996156.fastq.gz">, <Samples a342d2287fe4438f: "FS38996183.fastq.g
z">, <Samples 76376a4abda74fe1: "FS68624231.fastq.gz">]
```

In [18]:

```python
genera = samples.plot_heatmap(top_n=10,
    normalize= 'auto', rank='genus', title='Top 10 Genera',
    ylabel='Normalized Read Count', return_chart=True, legend="", label='Status2'
)
phyla = samples.plot_heatmap(top_n=10,
    normalize= 'auto', rank='phylum', title='Top 10 Phyla',
    ylabel='Normalized Read Count', return_chart=True, legend="", label='Status2'
)

genera | phyla
```

Out[18]:

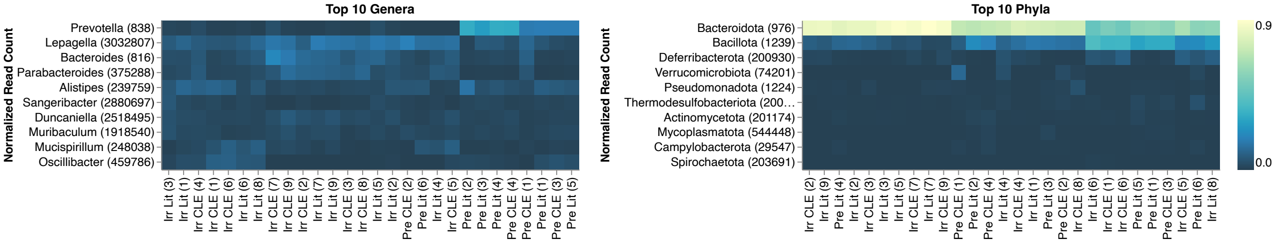

In [17]:

```python
def sort_by_status2(sample_labels):
    # Define your desired Status2 order
    status_order = ["Pre Lit", "Pre CLE", "Irr Lit", "Irr CLE"]

    # Extract the status part (before the parentheses) and sort accordingly
    def get_status_order(label):
        # Extract the status part before the parentheses
        status = label.split(' (')[0]  # e.g., 'Irr CLE (5)' -> 'Irr CLE'
        return status_order.index(status) if status in status_order else len(stat

    return sorted(sample_labels, key=get_status_order)

genera = samples.plot_heatmap(top_n=10,
    normalize='auto', rank='genus', title='Top 10 Genera', sort_x=sort_by_status2
    ylabel='Normalized Read Count', return_chart=True, legend="", label='Status2'
)

genera
```

Out[17]:

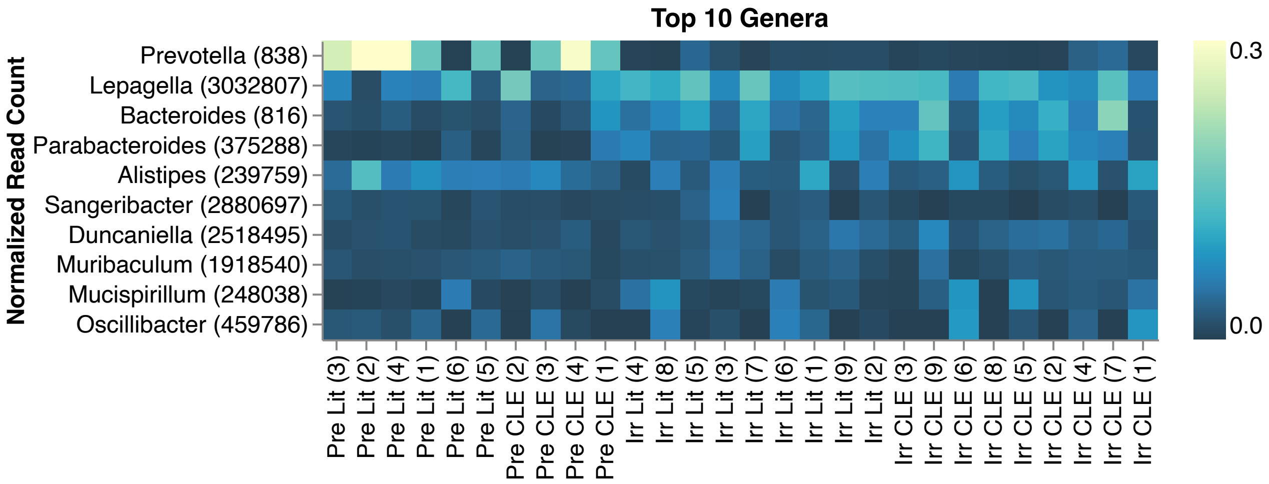

In [22]:

```
genera.data
```

Out[22]:

	classification_id	tax_id	Relative Abundance	Label	tax_name	
**0**	99bce79f5f9e46c4	3032807	0.110574	Irr Lit (1)	Lepagella (3032807)	https://app.onecodex.com/classif
**1**	e95a62fa8fa8421c	3032807	0.156832	Irr Lit (2)	Lepagella (3032807)	https://app.onecodex.com/classifi
**2**	1b5fb828156a45f1	3032807	0.066597	Pre Lit (1)	Lepagella (3032807)	https://app.onecodex.com/classif
**3**	fb7a978eba07450b	3032807	0.013353	Pre Lit (2)	Lepagella (3032807)	https://app.onecodex.com/classi
**4**	c1c3306bcaa242f4	3032807	0.069154	Irr CLE (1)	Lepagella (3032807)	https://app.onecodex.com/classif
**...**	...	...	...	...	...	
**275**	64ce2d6507f24d36	2880697	0.000000	Irr CLE (7)	Sangeribacter (2880697)	https://app.onecodex.com/classifi
**276**	47916150098d4399	2880697	0.016093	Irr Lit (8)	Sangeribacter (2880697)	https://app.onecodex.com/classif
**277**	5e474f22ddf44548	2880697	0.000000	Irr Lit (9)	Sangeribacter (2880697)	https://app.onecodex.com/classif
**278**	6674e09cac224504	2880697	0.008679	Irr CLE (8)	Sangeribacter (2880697)	https://app.onecodex.com/classifi
**279**	d49be622f7c4440c	2880697	0.000000	Irr CLE (9)	Sangeribacter (2880697)	https://app.onecodex.com/classifi

280 rows × 6 columns

Question #5: How do samples cluster?

First up, we'll plot a heatmap of weighted UniFrac distance between the first 30 samples in the dataset. This requires unnormalized read counts, so we'll generate a new, unnormalized dataframe.

In [24]:
```
# generate a dataframe containing read counts
# df_abs = samples.to_df(normalize=False)

# df_abs[:30].ocx.plot_distance(metric="weighted_unifrac")
```

In [25]:
```
help(samples.plot_pca)
```

```
Help on method plot_pca in module onecodex.viz._pca:

plot_pca(rank=<Rank.Auto: 'auto'>, normalize='auto', org_vectors=0, org_vectors_s
cale=None, title=None, xlabel=None, ylabel=None, color=None, size=None, tooltip=N
one, return_chart=False, label=None, mark_size=100, width=None, height=None, matc
h_taxonomy=True) method of onecodex.models.collection.SampleCollection instance
    Perform principal component analysis and plot first two axes.

    Parameters
    ----------
    rank : {'auto', 'kingdom', 'phylum', 'class', 'order', 'family', 'genus', 'sp
ecies'}, optional
        Analysis will be restricted to abundances of taxa at the specified level.
    normalize : 'auto' or `bool`, optional
        Convert read counts to relative abundances such that each sample sums to
1.0. Setting
        'auto' will choose automatically based on the data.
    org_vectors : `int`, optional
        Plot this many of the top-contributing eigenvectors from the PCA results.
    org_vectors_scale : `float`, optional
        Multiply the length of the lines representing the eigenvectors by this co
nstant.
    title : `string`, optional
        Text label at the top of the plot.
    xlabel : `string`, optional
        Text label along the horizontal axis.
    ylabel : `string`, optional
        Text label along the vertical axis.
    size : `string` or `tuple`, optional
        A string or a tuple containing strings representing metadata fields. The
size of points
        in the resulting plot will change based on the metadata associated with e
ach sample.
    color : `string` or `tuple`, optional
        A string or a tuple containing strings representing metadata fields. The
color of points
        in the resulting plot will change based on the metadata associated with e
ach sample.
    tooltip : `string` or `list`, optional
        A string or list containing strings representing metadata fields. When a
point in the
        plot is hovered over, the value of the metadata associated with that samp
le will be
        displayed in a modal.
    label : `string` or `callable`, optional
        A metadata field (or function) used to label each analysis. If passing a
function, a
        dict containing the metadata for each analysis is passed as the first and
only
        positional argument. The callable function must return a string.
    mark_size: `int`, optional
        The size of the points in the scatter plot.
    match_taxonomy : `bool`, default=True
        Whether or not to consider taxonomic names when looking for metadata fiel
ds mapped to
        plot attributes including `tooltip`, `color`, `label`, & `size`

    Examples
    --------
    Perform PCA on relative abundances at the species-level and color the resulti
```

```
ng points by
    'geo_loc_name', a metadata field representing the geographical origin of each
sample.

    >>> samples.plot_pca(rank='species', normalize=True, color='geo_loc_name')

    Change the size of each point in the plot based on the abundance of Bacteroid
es.

    >>> samples.plot_pca(size='Bacteroides')

    Display the abundances of Bacteroides, Prevotella, and Bifidobacterium in eac
h sample when
    hovering over points in the plot.

    >>> samples.plot_pca(tooltip=['Bacteroides', 'Prevotella', 'Bifidobacteriu
m'])
```

Question #6: Can I do PCA?

In [26]:
```
samples.plot_pca(color="Status2", title = 'PCA by Disease Status')
```

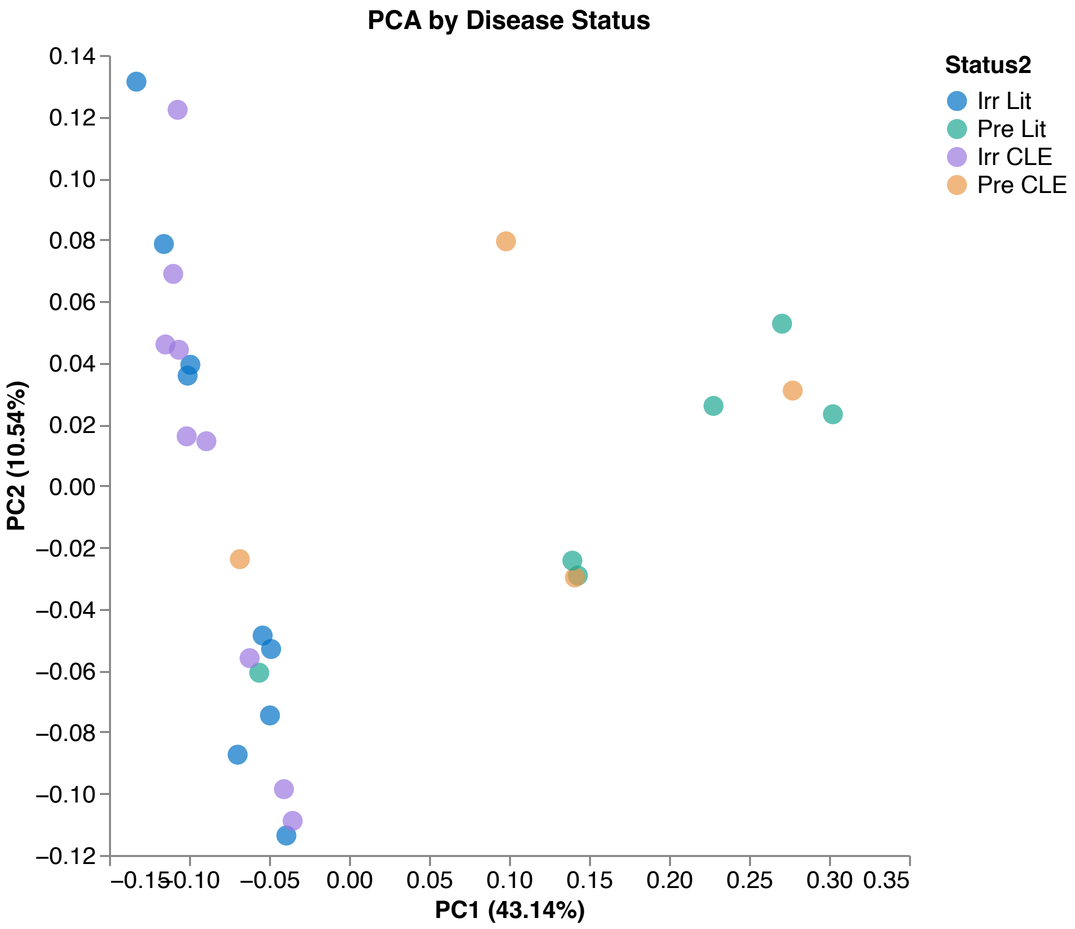

In [27]:
```
samples.plot_pca(color="Inject/non")
```

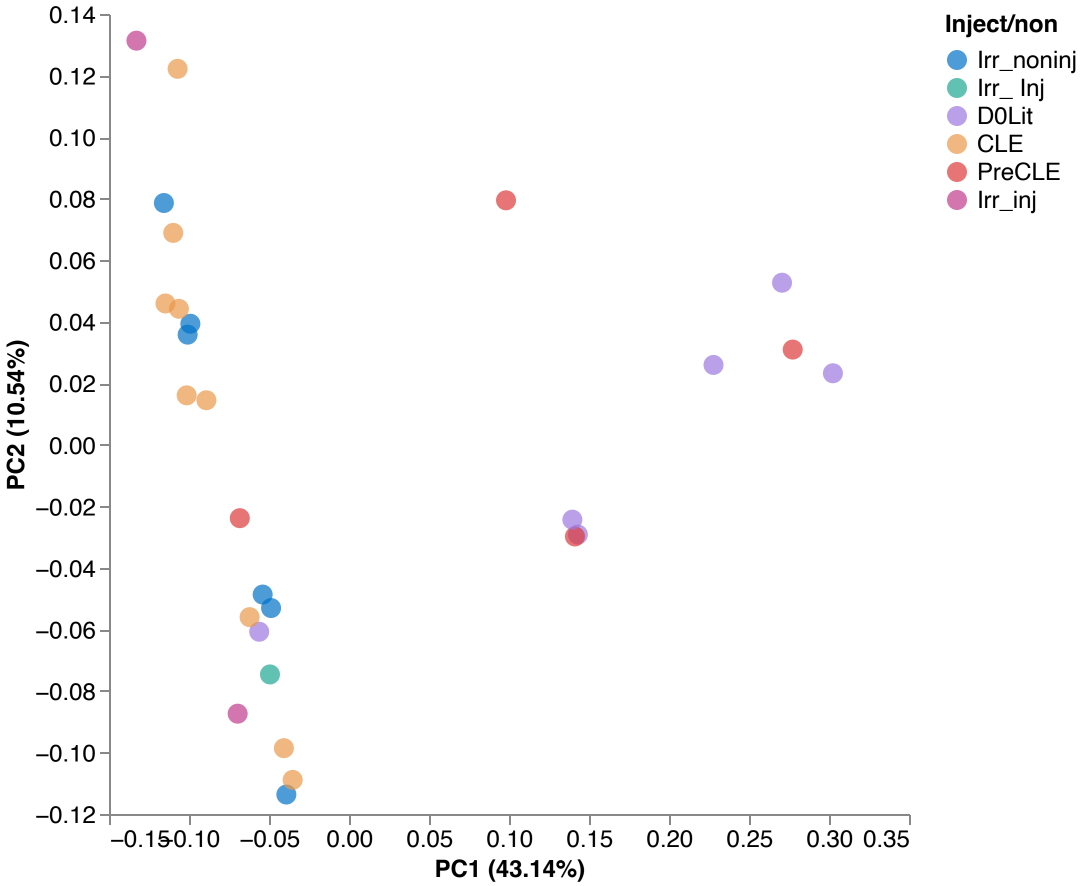

In [28]:

```
samples.plot_pca(color="Cage", title = 'PCA by Cage Number')
#edited to discrete variables in Vega editor
```

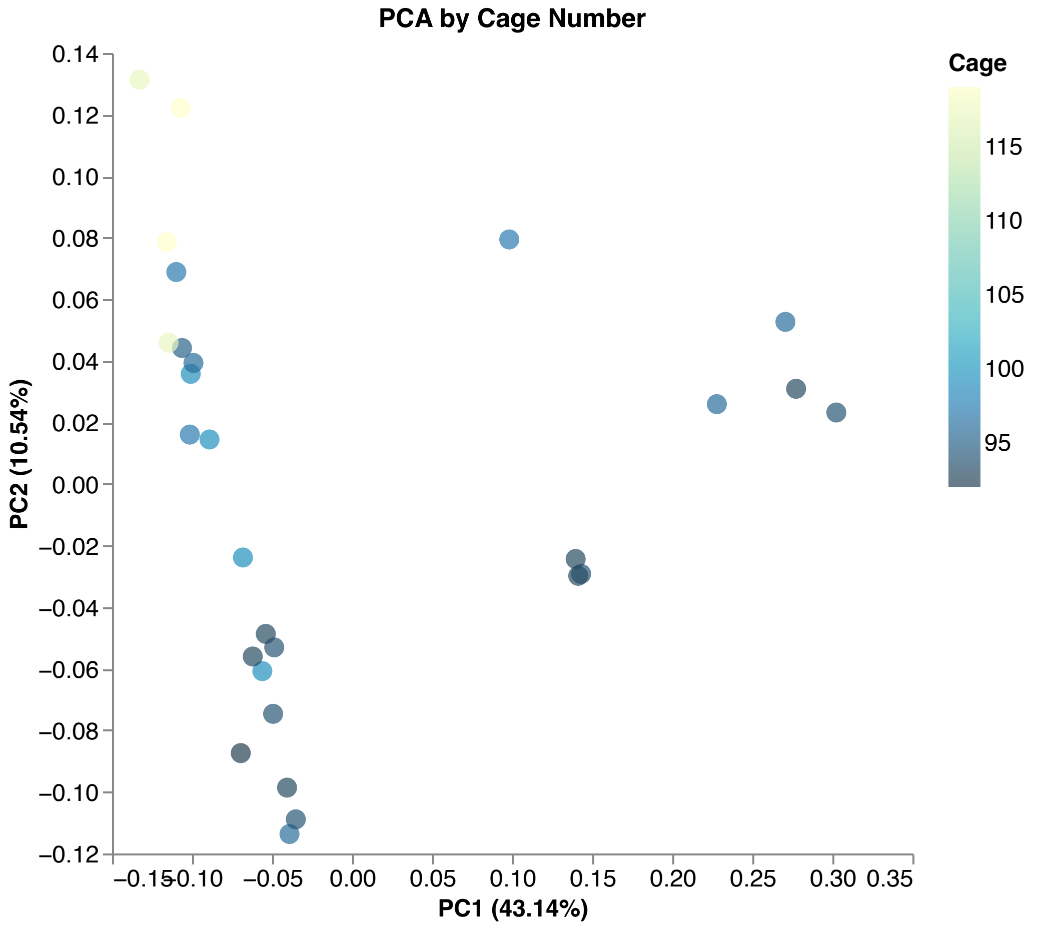

In [29]:

```
samples.plot_pca(color="Sex", title = 'PCA by Sex')
```

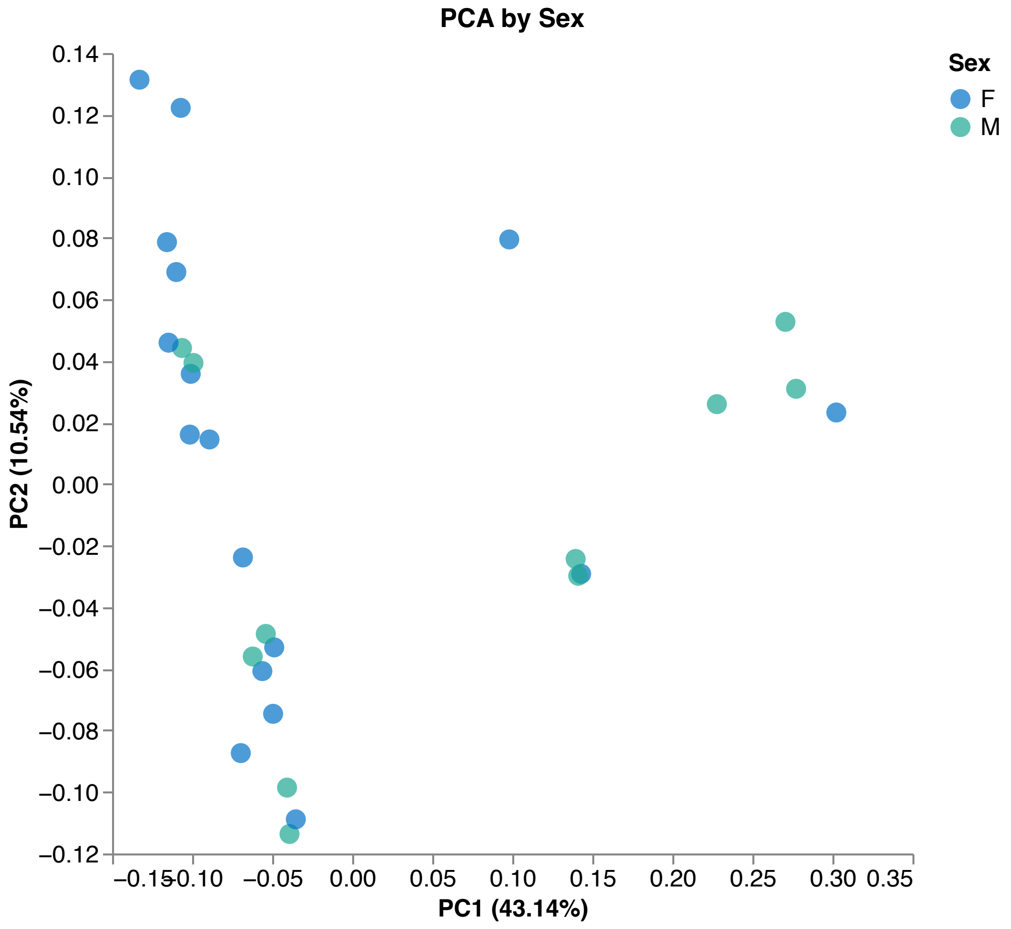

MDS

In [30]:
```
samples.plot_mds(
    metric="weighted_unifrac", method="pcoa", color="geo_loc_name"
)
```

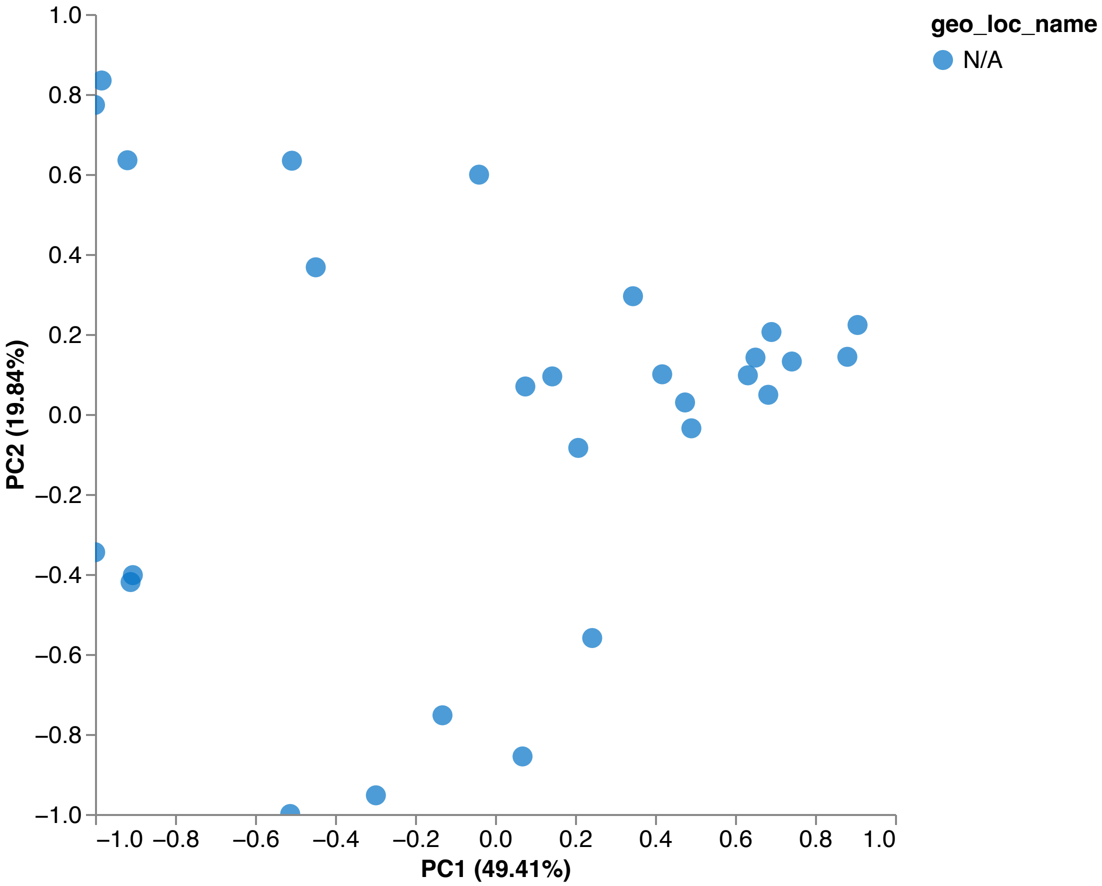
geo_loc_name
N/A
PC2 (19.84%)
PC1 (49.41%)
1.0
0.8
0.6
0.4
0.2
0.0
−0.2
−0.4
−0.6
−0.8
−1.0
−1.0 −0.8 −0.6 −0.4 −0.2 0.0 0.2 0.4 0.6 0.8 1.0

```
In [ ]:
```
